# Survey of five major grapevine viruses infecting Blatina and Žilavka cultivars in Bosnia and Herzegovina

**DOI:** 10.1371/journal.pone.0245959

**Published:** 2021-01-22

**Authors:** Ana Crnogorac, Stefano Panno, Ana Mandić, Mladen Gašpar, Andrea Giovanni Caruso, Emanuela Noris, Salvatore Davino, Slavica Matić

**Affiliations:** 1 Federal Agromediterranean Institute of Mostar, Mostar, Bosnia and Herzegovina; 2 Department of Agricultural, Food and Forest Sciences, University of Palermo, Palermo, Italy; 3 Department of Biological, Chemical and Pharmaceutical Sciences and Technologies, University of Palermo, Palermo, Italy; 4 Faculty of Agriculture and Food Technology, University of Mostar, Mostar, Bosnia and Herzegovina; 5 Institute for Sustainable Plant Protection, National Research Council of Italy (IPSP-CNR), Torino, Italy; Washington State University, UNITED STATES

## Abstract

The sanitary status of grapevines has not yet been considered sufficiently in vineyards throughout Bosnia and Herzegovina (BiH). An extensive survey of five major grapevine viruses in the country was carried out in 2019. A total of 630 samples from the two dominant autochthonous cultivars, named Žilavka and Blatina, were tested by DAS-ELISA for the presence of grapevine leafroll-associated viruses (GLRaV-1 and 3), grapevine fleck virus (GFkV), grapevine fanleaf virus (GFLV) and Arabis mosaic virus (ArMV). Eighty-eight % of the samples were positive for at least one virus, and all five viruses were detected, thought with different incidence, i.e. GLRaV-3 (84%), GFLV (43%), GLRaV-1 (14%), GFkV (10%) and ArMV (0.2%). The majority of infected plants (about 75%) were asymptomatic. Specific virus symptoms were observed in the remaining infected plants, together with the reported GLRaV vectors, *Planococcus ficus* and *Parthenolecanium corni*, while nematodes of the *Xiphinema* genus were not found in the GFLV- or ArMV-infected vineyards. The GLRaV-3 *CP* phylogenetic analyses showed 75–100% nucleotide identity between the BiH and reference isolates, and the BiH isolates clustered into the major group. The dNS/dS ratio indicated a negative selection of the virus population, and the lack of geographical structuring within the population was observed. In addition, putative GLRaV-3 recombinants with breakpoints in the 5’ of the *CP* gene were detected, while no recombinant strains were identified for the other four viruses. The obtained results indicate a deteriorated sanitary status of the cultivated grapevines, the prevalence and intraspecies genetic diversity of GLRaV-3 throughout the country. The establishment of certified grapevine material and adequate virus vector control is therefore of primary importance to prevent further spread of these viruses. This study presents the results of the first molecular characterisation of grapevine viruses in Bosnia and Herzegovina.

## Introduction

Grapevine harvested areas throughout the world cover more than 7 million ha, around half of which is located in Europe, which ranks first in grapevine production [1]. Grapevine is an economically important crop in the Balkan-Mediterranean area, and it ranks third among the cultivated fruit crops in Bosnia and Herzegovina (BiH). Almost 4,500 ha is used for the cultivation of grapevines in the country, with an annual production of around 40,000 tons of grapes in 2019 [1]. The grapevine production in BiH is concentrated in two main vineyard regions, Lištica and Mostar, both located in the Southern part of the country (Herzegovina). Grapevine cultivation in these regions is mainly represented by autochthonous cultivars (cvs.). Two of the most important and commercial cultivars are the white grapevine ’Žilavka’ and the red grapevine ’Blatina’, which have been grown in these regions since the 14^th^ century [2,3]. These two cultivars contribute predominantly to the whole wine production of the country (227,500 hl; Mario Leko, personal communication).

Viruses represent the most dangerous and economically important pathogens of grapevine, and 86 distinct virus species belonging to 17 families and 34 genera have been identified in this crop [4,5]. Viral disease management is often difficult, mainly due to the absence of effective chemical compounds for their control, the rapid evolution of the viruses through mutation and genetic recombination [6–8], their easy adaptation to the local environmental conditions and the breakdown of plant genetic resistance [9]. Therefore, it is very important to know the genetic diversity and dispersion modes of these pathogens, especially in a perspective ecologically sustainable environment [10].

Grapevine leafroll disease (GLD) is the most widespread and economically important grapevine virus disease worldwide, and it is associated with a complex of viruses known as grapevine leafroll-associated viruses (GLRaVs), which belong to the *Closteroviridae* family (genera *Ampelovirus*, *Closterovirus* and *Velarivirus*) [11]. Grapevine leafroll-associated virus 3 (GLRaV-3) is the most important species within this virus complex and the most predominant virus pathogen of grapevines throughout the world [12–15]. Other economically important and widespread viruses in the Mediterranean region include grapevine leafroll-associated viruses 1 and 2 (GLRaV-1, -2) (other viruses of the GLD complex), grapevine fleck virus (GFkV), grapevine virus A, and grapevine fanleaf virus (GFLV) [16–20].

GFkV belongs to the *Tymoviridae* family (genus *Maculavirus*), and it is associated with grapevine fleck disease. *Vitis vinifera* cultivars infected by GFkV are symptomless, but the virus causes leaf deformation, vein clearing and a vegetative growth reduction in the sensitive indicator *Vitis rupestris* cv. ’St. George’ [5,19].

GFLV, belonging to the *Secoviridae* family (genus *Nepovirus*), is the most widespread and the main causative agent of grapevine fanleaf degeneration disease (GFDD). Another nepovirus associated with GFDD, although to a lesser extent, is Arabis mosaic virus (ArMV) [21]. The main symptoms of the disease are distorted leaves, yellow mosaic stripes, vein clearing and banding, zigzag growth, double nodes, and short/deformed internodes; the disease might also cause the decline and destruction of vinestocks [22,23].

Grapevines may also be infected with viruses without displaying any noticeable symptoms (latent infection) [20,24,25]. Therefore, it is recommended to take samples from both symptomatic and asymptomatic plants to perform virus testing [24]. The correlation of virus infections and symptom expression might be related to various factors, such as environmental conditions, virus strain, or multiple pathogen infections [26].

Grapevine viruses can be transmitted through grafting and by vectors. The current lack of virus-free propagation material of autochthonous cultivars in BiH may enhance the relevance of graft transmission in the spread of grapevine viruses [20]. Many authors have reported mealybugs and soft scale insects (order *Hemiptera*), as being the vectors of GLRaV-3 and GLRaV-1 [14,27–30]. Dimitrijevic [31] reported the spread of leafroll in vineyards throughout the former Yugoslavia and associated it with the presence of scale insect populations. GFLV can be transmitted by the ectoparasitic nematode *Xiphinema index* [17,32,33], whose major host is a domesticated grapevine (*V*. *vinifera* subsp. *vinifera*) [34]. ArMV is transmitted by another ectoparasitic soilborne nematode, *Xiphinema diversicaudatum* [35]. The primary spread of GFkV is known to occur through infected propagation material and no vector has yet been reported for this virus [36].

Upon virus infection, grapevine plants remain infected throughout their life span, and prevention of infection includes the production of certified virus-free and virus-tested plant material. Most vineyards in BiH have been established with *Conformitas Agraria Comunitatis* (CAC) category of propagation material for autochthonous cultivars. To improve the sanitary status of such grapevines, a decade ago the Federal Agro-Mediterranean Institute of Mostar started a mass selection of the most important cultivars. The selection process involved screening for the presence of five major grapevine viruses, that is GLRaV-1, GLRaV-3, GFkV, GFLV and ArMV, which pursuant to the legislation in the country [37] are to be subjected to mandatory testing for the propagation material. Therefore, the main objective of this study was to monitor for the presence of these five viruses, GLRaV-1, GLRaV-3, GFkV, GFLV and ArMV, in symptomatic and asymptomatic samples derived from BiH commercial vineyards enrolled in local nursery production and used for the propagation of grapevine material. Two other objectives were to perform a molecular characterization of the detected viruses and to investigate the presence of grapevine virus vectors.

## Materials and methods

### Field survey and sample collection

In order to investigate the sanitary status related to virus infections of the local grapevine cultivars, 278 samples of the cv. Žilavka and 352 samples of the cv. Blatina were collected during late summer in 2019. Field surveys were carried out in 8 commercial vineyards: Višići [collection vineyard, Čapljina (ČA)], Blizanci [Hercegovina vino doo., Čitluk (ČI)], Lopate [Hercegovina vino doo., Kosor, Mostar (MO)], Plantaže-Otok [Erovin doo., Ljubuški (LJB)], Dugolaza-Ražovina (vinarija Nuići doo., Crnopod, LJB), Sovići [private vineyard, Grude (GR)], Buna Stup (private vineyard, MO) and Poprati [private vineyard, Stolac (ST)]. These vineyards are involved in the preparation of local nursery material and in the production of fresh grapes and wine. The field study (number 19_SG) was carried out under permission of the Federal Agromediterranean Institute of Mostar, Mostar, Bosnia and Herzegovina.

A total of 630 samples were collected from 13 to 20-year old Žilavka and Blatina plants grafted onto the Kober 5BB rootstock (*Vitis berlandieri* x *Vitis riparia*). A dormant cutting sample of each plant was collected from the basal part of the scion. Each sample was divided into two subsamples (three replicates each) to carry out serological and molecular tests. Both symptomatic and asymptomatic plants were sampled randomly and geo-referenced by means of GPS using the Planthology mobile application [7]. Simple random sampling method based on data of vineyard planting area with 99% confidence interval [38,39] was used to select the sampling points in vineyards, covering 36 vineyards blocks in eight orchards. The field survey also involved a symptomatology assessment and the search for the presence of virus vectors in the vineyards.

### Serological test

All the samples were analysed by means of double antibody sandwich enzyme-linked immunosorbent assay (DAS-ELISA) [40] using specific antibodies to GLRaV-1, GLRaV-3, GFLV, GFkV and ArMV (Bioreba AG, Reinach BL 1, Switzerland). Three-hundred mg of cortical scrapings of each sample was mixed and homogenised with 0.5 ml extraction buffer (0.5 M Tris-HCl, 2% PVP-24, 1% PEG 6000, 0.14 M NaCl, 0.05% Tween 20, pH 8.2) and a 1:10 dilution (w/v) of each sample was used for DAS-ELISA, following the manufacturer’s instructions. Lyophilized plant tissue infected by GLRaV-1, GLRaV-3, GFLV, GFkV and ArMV (Bioreba AG), and healthy plant tissue (Bioreba AG) were used as positive and negative controls, respectively. Optical densities (O.D.) were measured at 405 nm, using a Sunrise microplate reader (Tecan, Männedorf, Switzerland) 2 h after the addition of the p-nitro-phenylphosphate substrate. The sample was considered positive if its OD_405_ value was at least twice the negative control value.

### Soil analyses for the presence of nematodes

Soil samples from vineyards in which the nematode transmitted viruses (GFLV and ArMV) were detected by DAS-ELISA were collected and analysed for the presence of *X*. *index* and *X*. *diversicaudatum*, respectively. Soil samples (ca. 250 g) were investigated for the presence of nematodes using the sieving method, as described by Flegg [41].

### RNA extraction

Eighty-one randomly selected samples of Žilavka and Blatina cvs. reacting positively in DAS-ELISA were also tested by means of a multiplex RT-PCR assay. The samples were ground into powder with a mortar and pestle in liquid nitrogen and stored at -20°C for RNA isolation. Lyophilized plant tissue infected by GLRaV-1, GLRaV-3, GFLV, GFkV and ArMV as positive control (Bioreba AG), as well as negative controls (healthy plant tissue; Bioreba AG) were subjected to RNA extraction. Total RNA was extracted using an RNeasy Plant Mini kit (Qiagen, Hilden, Germany), according to the manufacturer’s instructions, and eluted in 50 μl RNase-free water. The obtained total RNA was re-suspended in 50 μl of RNase-free water, and the RNA concentration was measured twice with a UV-Vis Nanodrop 1000 spectrophotometer (Thermo Fisher Scientific, Waltham, USA), adjusted to approximately 50 ng/μl and stored at -80°C until use.

### Multiplex RT-PCR

One-step multiplex RT-PCR was carried out as described by Gambino and Gribaudo [42]. The assay was performed with a OneStep RT-PCR Kit (Qiagen) in a 25 μl mixture containing 100 ng of total RNA treated with the Ambion^™^ DNase I (Thermo Fisher Scientific), according to the manufacturer’s instructions, 1× RT-PCR buffer (Qiagen), 0.4 mM dNTPs, 0.5 μl of each primer, and 2 μl of a OneStep RT-PCR enzyme mix (Qiagen). Primers were used at final concentrations of 1.2 μM for GLRaV-3, 0.3 μM for GLRaV-1, 0.2 μM for GFkV, 1.6 μM for GFLV, and 2 μM for ArMV. The cycling conditions were as follows: reverse transcription at 47°C for 50 min, initial denaturation at 94°C for 5 min, followed by 35 cycles of 94°C for 30 s, 50°C for 1 min and 72°C for 90 s, and a final extension at 72°C for 5 min. The PCR products were electrophoresed on 1.5% agarose gel, pre-stained with GelRed^®^ Nucleic Acid Gel Stain (1×, Biotium, Hayward, USA), and visualised by means of UV light. In order to ensure the absence of any nonspecific amplification, each RT-PCR run included molecular grade water and a healthy plant sample as negative controls. After the identification of single/mixed infections, RT-PCR assays specific for each of the detected viruses were also carried out on randomly selected samples infected by GLRaV-3 (35), GLRaV-1 (4), GFLV (1), and ArMV (1). In addition, the full-length coat protein (*CP*) gene of GLRaV-3 and portion of gene sequences of the other three viruses, i.e. partial *CP* genes for GLRaV-1 and GFLV and the polyprotein gene for ArMV were amplified by RT-PCR, using the conditions and primers listed in S1 Table. Four of the GFkV isolates already had sequences available in the replicase gene [43], which were used for the subsequent phylogenetic analyses.

### Sequence analyses

Amplified PCR products of GLRaV-3, GLRaV-1, GFLV and ArMV isolates were purified using a QIAquick PCR purification kit (Qiagen), following the manufacturer’s instructions. The purified PCR products were sequenced in both directions at BMR Genomics (Padua, Italy). The full-length CP sequences of 35 GLRaV-3, partial *CP* gene sequences of four GLRaV-1 and one GFLV isolate, and partial polyprotein gene sequence of one ArMV isolate were deposited in GenBank under Accession Numbers: MT432352-MT432386, MK526895-MK526898, MW147746, and MW413756, respectively (S1 File). Comparison with sequences available in the GenBank database was performed using the BLAST algorithm (www.ncbi.nlm.nih.gov).

The CLUSTALW program [44] was used to construct a multiple nucleotide (nt) sequence alignment. Phylogenetic relationships were inferred by means of the maximum likelihood estimation method based on the Kimura 2-parameter with 1,000 bootstrap replicates [45,46], and all the analyses were performed using the MEGA X program [47].

The role of natural selection in the evolution of local GLRaV-3 isolates was studied at the molecular level by evaluating the rate of synonymous substitutions per synonymous site (dS) and the rate of nonsynonymous substitutions per nonsynonymous site (dN). These and other genetic diversity parameters were determined by means of DNA Sequence Polymorphism v. 6 software [48]. These values were not calculated for GLRaV-1, GFLV and GFkV due to the limited number of sequenced isolates.

The alignments of the all the sequenced isolates were analyzed, together with the available reference isolates using the RDP4 (v.4.39) software package to detect any potential recombination events [49]. RDP4 parameters were set as default values.

## Results

### Field survey

**Fig 1:**
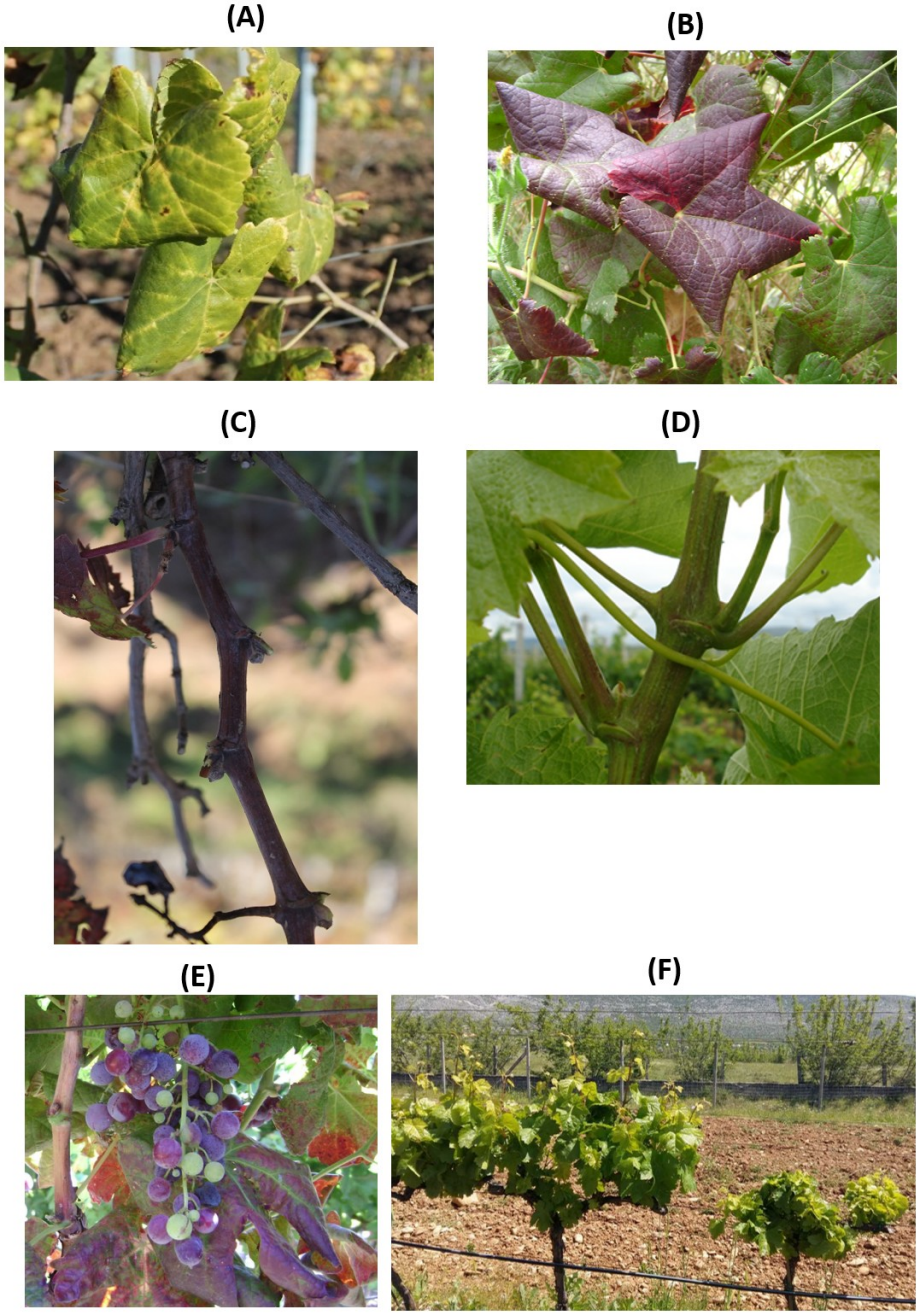
Symptomatology observed on infected grapevine plants surveyed in this work. (A) Leafroll and yellowing (cv. Žilavka) associated with grapevine leafroll-associated virus 3 (GLRaV-3). (B) Leafroll and reddening (cv. Blatina) associated with GLRaV-3. (C) Zig-zag growth associated with grapevine fanleaf virus (GFLV). (D) Short internodes associated with GFLV and GLRaV-3. (E) Uneven berry ripening associated with GLRaV-3. (F) Reduced vigour of the plant associated with grapevine fleck virus (GFkV) and GLRaV-3.

The main symptoms observed during the field survey consisted in downward rolling of leaves followed by reddening (cv. Blatina) or yellowing (cv. Žilavka) of the leaf tissue between the major veins (Fig 1). Leaf-rolling and leaf colour change were observed in all the plants with single (GLRaV-1 or GLRaV-3), double and triple infections (with presence of one or both, GLRaV-1 or GLRaV-3), subsequently detected by DAS-ELISA. Delayed vegetation as well as distorted and asymmetrical leaves were observed in plants with single or mixed infections involving GFLV. Zigzag growth was observed in cv. Blatina, while shortening of the internodes was observed in both cultivars infected by single and mixed GFLV infections. The symptoms detected in the unique plant infected by ArMV consisted in leaf yellowing, while no symptoms were visible in plants with single GFkV infection. Uneven berry ripening was observed in plants infected only by GLRaV-3, whereas delayed ripening was found in the mixed GLRaV-3 + GFLV and GLRaV-1 + GLRaV-3 + GFLV infections. Reduced vigour of the plants and smaller clusters with an increased number of berries were observed in the mixed GFkV + GLRaV-3 and GFkV + GLRaV-3 + GFLV infections (Fig 1). Notably, most (75%) of the surveyed plants were asymptomatic since the vineyards surveyed are used for local nursery production.

**Fig 2:**
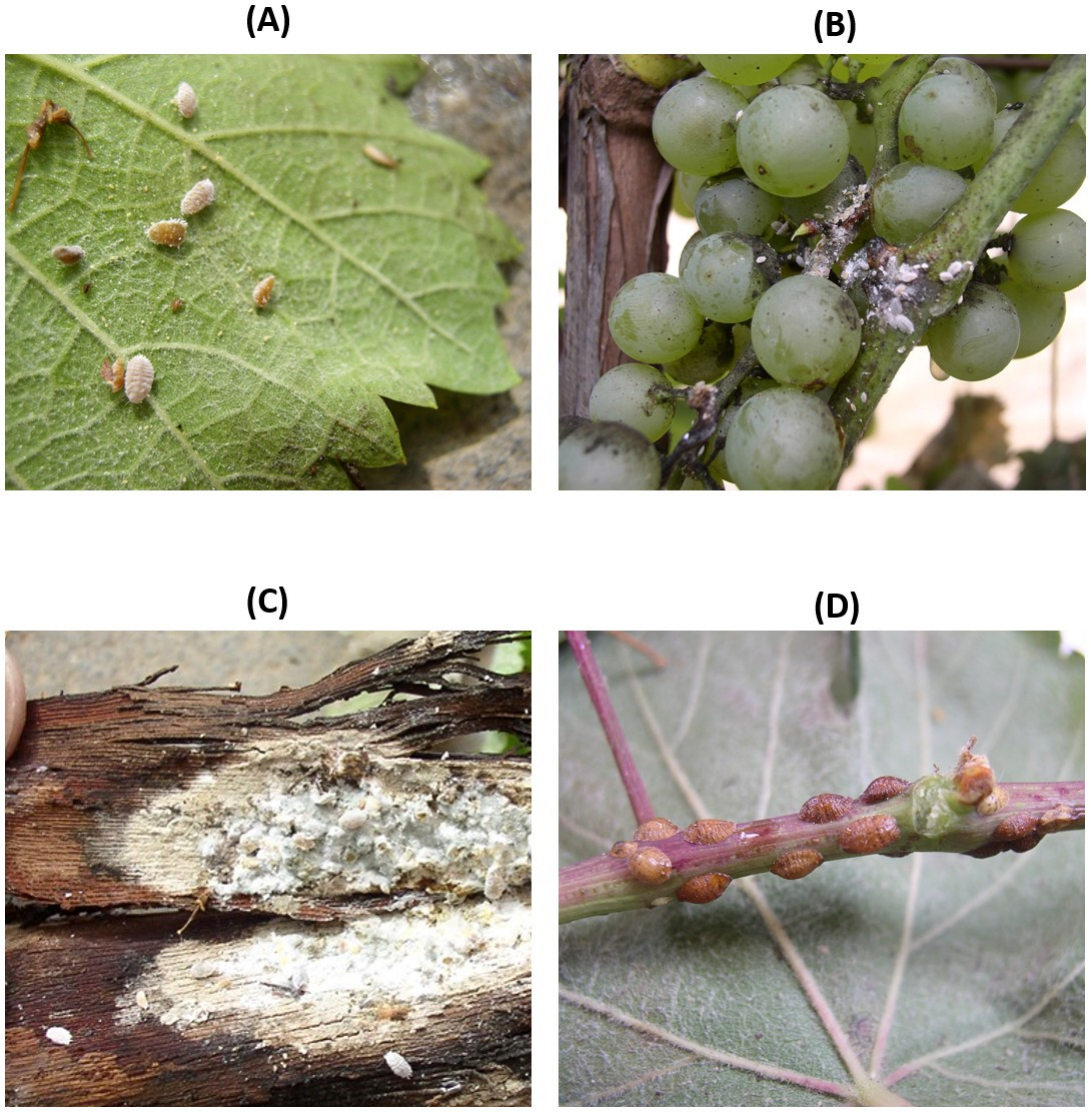
Mealybugs and scale insects observed during the field survey on grapevine leafroll-associated virus (GLRaV)-infected grapevine plants. (A-B) Nymphs and adults of *Planococcus ficus*. (C) Nymphs and adults of *Parthenolecanium corni*. (D) Adults of *P*. *corni*.

The vineyards were also inspected for the presence of mealybugs and soft scale insects, the known vectors of GLRaVs [50,51]. Although the vineyards had been treated with insecticides against these pests, a few mealybugs and soft scale insects were observed. The following species were identified by morphological and morphometric methods [52–54]: *Planococcus ficus* (Signoret), the Mediterranean vine mealybug (*Pseudococcidae* family), and the scale insects *Parthenolecanium corni* (Bouché; the European fruit lecanium), *Parthenolecanium persicae* (Fabricius; the European peach scale), *Pulvinaria vitis* (Linnaeus; the cottony grape scale), and *Neopulvinaria innumerabilis* (Rathvon; the cottony maple scale), all of which have already been reported in other countries as vectors of GRLaV-1 and -3 [28,55–57]. Among the identified species, the most dominant ones were *P*. *ficus* and *P*. *corni* (Fig 2).

### Serological test

A total of 278 samples of Žilavka and 352 of Blatina from 8 commercial vineyards were investigated for the presence of five major grapevine viruses. Five hundred and fifty-three (88%) out of the 630 samples, gave positive result for at least one virus (Table 1). Specifically, the detected viruses were: GLRaV-3 (84%), GFLV (43%), GLRaV-1 (14%), GFkV (10%) and ArMV (0.2%) (Table 2). In both cultivars Žilavka and Blatina, GLRaV-3 was the most represented virus, in both single and mixed infections. Mixed infections contributed by 45% (250 samples) of the total infections, with a higher presence in Blatina (55%, 168 samples) than in Žilavka (33%, 82 samples). Double infections of GLRaV-1 + GLRaV-3, GLRaV-3 + GFLV and GLRaV-3 + GFkV, and triple infections of GLRaV-1 + GLRaV-3 + GFLV, GLRaV-1 + GLRaV-3 + GFkV and GLRaV-3 + GFkV + GFLV were identified in both cultivars (S2 and S3 Tables). The most frequent mixed infection was the double infection of GLRaV-3 + GFLV present in 14% of cv. Žilavka samples (S2 Table) and 26% of cv. Blatina (S3 Table). Among the triple infections, the most prevalent were GLRaV-1 + GLRaV-3 + GFkV in cv. Žilavka (3%; S2 Table) and GLRaV-3 + GFLV + GFkV in cv. Blatina (13%; S3 Table).

**Table 1:** Grapevine virus infection rate detected by double antibody sandwich ELISA (DAS-ELISA) in Žilavka and Blatina cultivars.

Cultivar	No. of samples		Infection rate (%)
	Tested	Infected	
Žilavka	278	246	88.49
Blatina	352	307	87.21
**Total**	630	553	87.77

**Table 2:** Relative incidence of grapevine viruses.

Cultivars	Number of infected samples	GLRaV-3	GFLV	GLRaV-1	GFkV	ArMV
Žilavka	246	223 (90.65)*	53 **(21.54)**	39 **(15.85)**	29 **(11.79)**	1
Blatina	307	242 **(78.83)**	183 **(59.61)**	37 **(12.05)**	25 **(22.80)**	0
Total	553	465	236	76	54	1
**Percentage (%)**		**84.09**	**42.67**	**13.74**	**9.77**	**0.18**

* number in brackets refers to the percentage.

**Fig 3:**
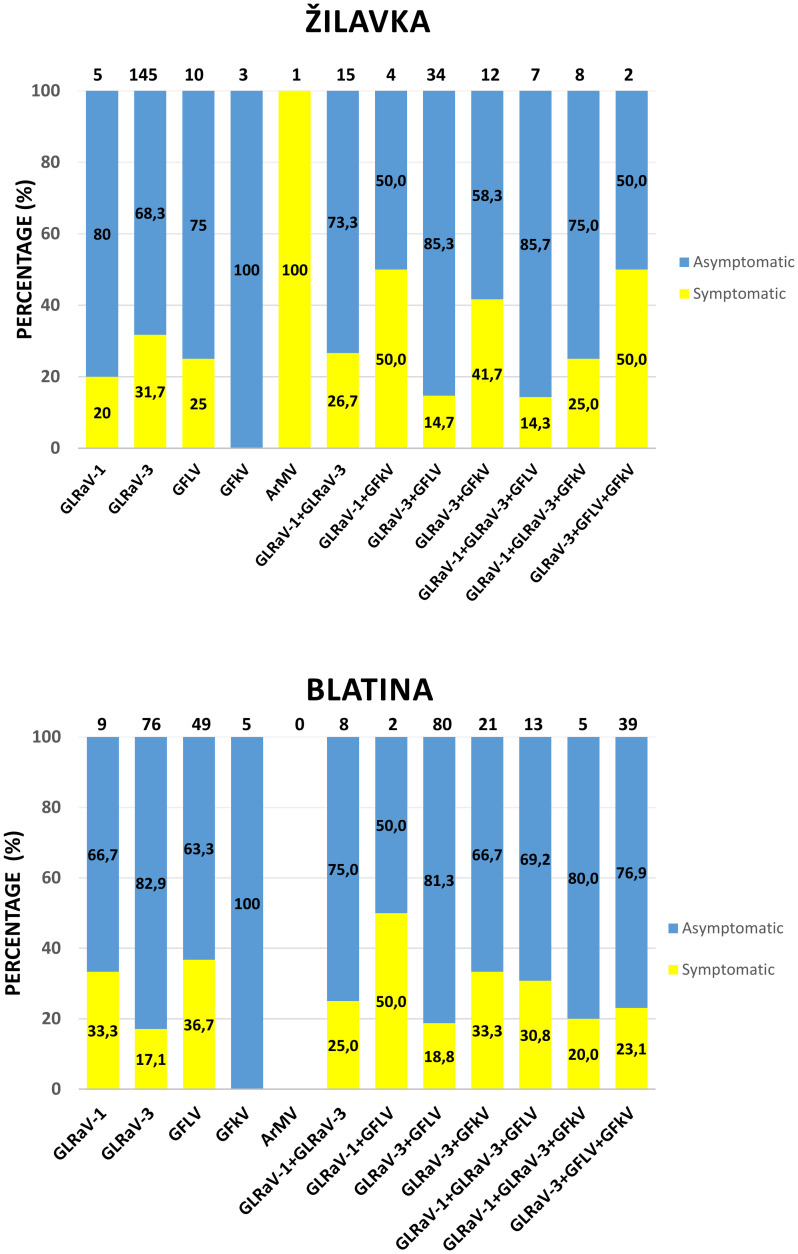
Comparative assessment of virus profiles of symptomatic *versus* asymptomatic infected plants in cvs. Žilavka and Blatina. Relative incidence of single and mixed viral infections is based on the double antibody sandwich ELISA (DAS-ELISA) results. Numbers on top of the columns indicate the numbers of infected samples.

Out of the infected plants, the majority were asymptomatic (72% in cv. Žilavka, and 76% in cv. Blatina). Furthermore, no important differences in virus incidence were observed between samples collected from symptomatic and asymptomatic grapevines (S2 and S3 Tables; Fig 3), i.e. all virus combinations (single and multiple) were detected in both symptomatic and asymptomatic plants, except for samples where only ArMV (symptomatic) or only GFkV (asymptomatic) were present (Fig 3).

### Soil analyses for the presence of nematodes

The presence of *X*. *index* and *X*. *diversicaudatum*, the nematode vectors of GFLV and ArMV, respectively [17,32,33,35], was not observed after using the sieving method in the soil samples collected from nepovirus-affected vineyards.

### Multiplex RT-PCR and sequence analyses of GLRaV-3 isolates

Based on DAS-ELISA results, 81 positive samples of cvs. Žilavka and Blatina were subjected to a multiplex RT-PCR. This technique confirmed the results of serological assays obtained for both single and mixed infections, without any additionally detected positive infections (S4 Table; S1 Fig).

**Fig 4:**
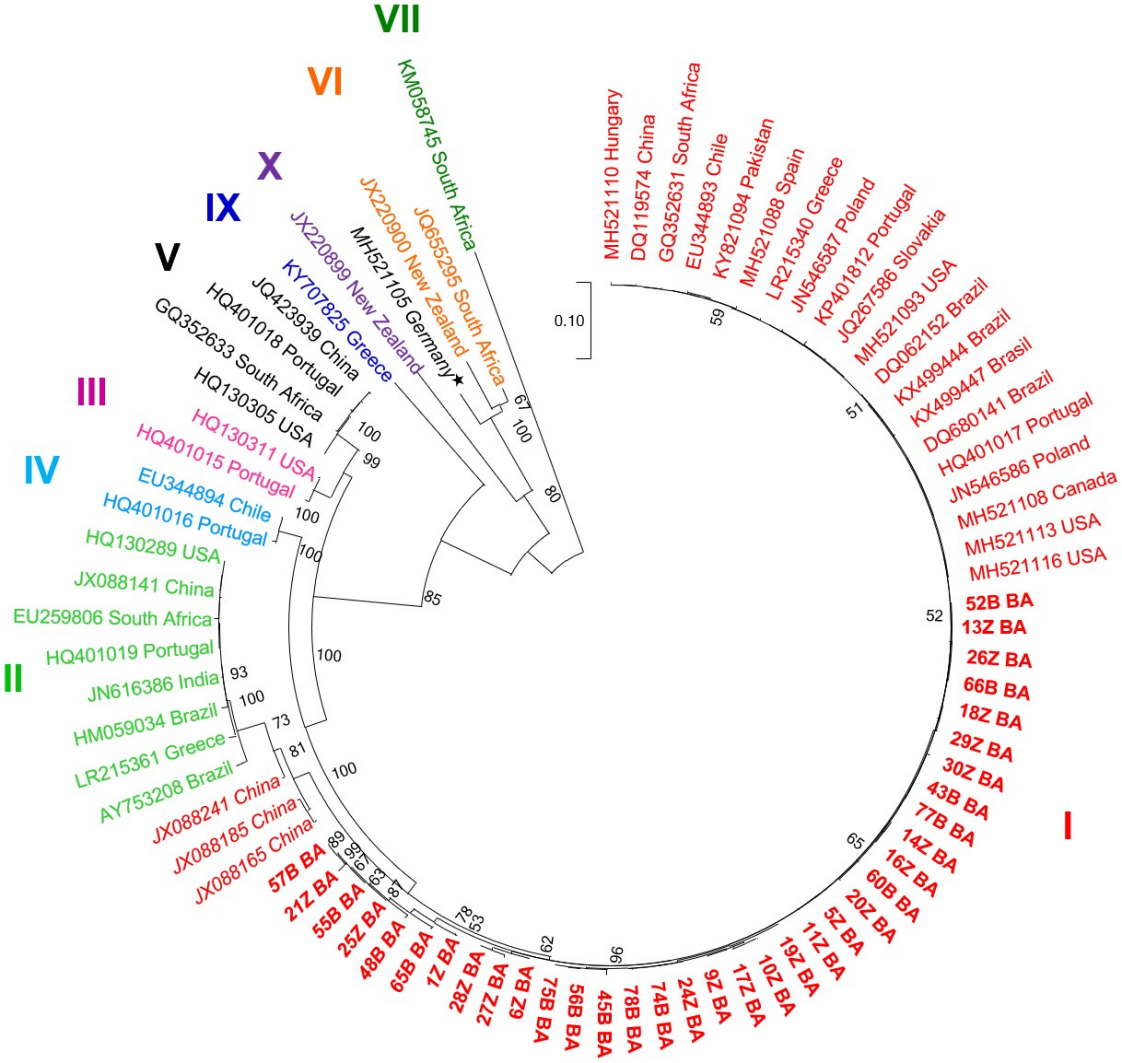
Phylogenetic relatedness of grapevine leafroll-associated virus 3 isolates obtained from full-length coat protein sequences, as inferred from a maximum likelihood analysis. Thirty-five BiH isolates (in bold) and forty-five reference isolates were included in the analysis. The accession number and the origin of each isolate are indicated. The bootstrap consensus tree was inferred from 1000 replicates and the bootstrap values of less than 50% are not presented. Phylogenetic groups are assigned as reported by Maree et al. [59] (groups I-VII) and Diaz-Lara et al. [60] (groups IX and X). Group VIII [59] was not included in phylogenetic analysis because it contained the sequences (JF723301-JF723378) retrieved from the NCBI. The isolate MH521105 (marked with a star) is not assigned to any of phylogenetic group [60], while the divergent isolates from this study and Farooq et al. [58] study (within the group I) are in italics.

The sequence comparisons and phylogenetic analyses of 35 randomly selected GLRaV-3 samples from the country confirmed that all the sequenced isolates belonged to this most prevalent virus (S1 File). The maximum likelihood phylogenetic tree including 35 GLRaV-3 BiH isolates and 45 reference grapevine isolates from 16 countries showed that they all clustered into 9 distinct phylogenetic groups (groups I-VII, IX and X; Fig 4). The 35 BiH isolates clustered into the major phylogroup (group I) that also included 20 reference isolates. These isolates originated from 14 countries from the Mediterranean Basin (Bosnia and Herzegovina, Spain, Portugal and Greece), Central-Northern Europe (Hungary, Slovakia and Poland), Central-Eastern Asia (China and Pakistan), North America (the USA, Canada), South America (Brazil and Chile) and South Africa. A minor subgroup within the group I contained the BiH isolates, 55B BA, 57B BA, 21Z BA, 25Z BA, 48B BA, 65B BA and 1Z BA (four of which were recombinants), and it was grouped next to the subgroup containing three Chinese divergent isolates (JX088241, JX088165 and JX088185) [58]. The other phylogroups (II-VII, IX and X) included the remaining reference isolates, from 9 countries throughout the world.

The pairwise percent identity of the BiH *CP* sequences ranged from 96.3–100% (nt) to 97.5–100% (amino acid, aa) (S2 Fig). When the CP sequences included both the BiH and reference isolates, the range was much lower; 75.2–100% (nt) and 87.58–100% (aa) (S3 Fig). The average genetic diversity of BiH GLRaV-3 was 0.010 for the full-length *CP* gene. The synonymous substitutions (dS) per site (0.3144) were higher than the nonsynonymous substitutions (dNS) (0.0386) and resulted in a dNS/dS ratio of 0.1230 (Table 3). These values indicate the hypothesis of negative or purifying selection. The overall nucleotide diversity (π) value for all the GLRaV-3 isolates was 0.0547, while a value of 0.0096 was found for the isolates from BiH. Tajima’s D, Fu and Li’s D, and Fu and Li’s F tests were negative for *CP*, thus confirming the occurrence of selective processes or population expansion.

**Table 3:** Genetic diversity parameters of the full-length coat protein sequences of GLRaV-3 isolates from Bosnia and Herzegovina.

Isolates	n	d	dS	dNS	dNS/dS	S	π	θ (S)	θ (π)	Tajima’s D	Fu and Li’s D	Fu and Li’s F
All	106	0.062	5.0086	0.3448	0.0688	422	0.0547	0.1135	0.0590	-1.8660	-2.3150	-2. 3870
BiH	35	0.010	0.3144	0.0386	0.1230	46	0.0096	0.0122	0.0097	-0.8584	-1.0716	-1.1000
Reference	71	0.057	4.6626	0.3123	0.0669	419	0.0718	0.1224	0.0794	- 1.5803	- 1.7228	-1.8135

n = number of isolates; d = diversity; dS = synonymous substitutions; dNS = non-synonymous substitutions; S = number of polymorphic sites; π = nucleotide diversity; BiH = Bosnia and Herzegovina.

The full-length BiH GLRaV-3 *CP* gene sequences were also investigated for the presence of recombination signals using RDP4 software. One putative recombination event involving the isolate 27Z BA was detected with break points at 180 and 239 nt. The isolate 65B BA shared the same recombination event (break points 180–318 nt), while three isolates (1Z BA, 48B BA, 55B BA) also showed traces of the same recombination event (break points 180–330 nt). The isolate 27Z BA had isolates IS2 (Brazil, HM059034) and 45B BA (BiH) as the potential major and minor parents, while the remaining recombinant isolates had isolates 45B BA and IS2 as the putative major and minor parents (Table 4).

**Table 4:** Recombination event in the coat protein coding region of 35 BiH and 71 reference GLRaV-3 isolates.

Recombinant isolate	Recombinant phylogenetic group*	Start position in *CP*	End position in *CP*	Parental Sequences*	Parental phylogenetic group	P-value by SiScan*
27Z BA	I	180	239	IS2 × 45B BA	II × I	4.724 × 10^−4^
65B BA	I		318	45B BA × IS2	I × II	
1Z BA, 48B BA, 55B BA	I		330	45B BA × IS2	I × II	

*Phylogenetic grouping reported by Maree et al. [59] and Diaz-Lara et al. [60].

### Phylogenetic analyses of GLRaV-1, GFLV, ArMV and GFkV

GLRaV-1 phylogenetic analyses, based on the partial *CP* gene sequences of four BiH isolates and 60 reference isolates from 14 countries, grouped the isolates into eight distinct groups. The BiH isolates clustered into three groups (I, II, and IV), together with reference isolates originating from different continents, while the other five groups included the remaining reference isolates (S4 Fig; S1 File).

GFLV phylogenetic analyses of the partial *CP* gene divided the studied isolates into two groups. The major group contained the isolates from different European countries, including the BiH 5Z BA isolate (S5 Fig; S1 File). ArMV phylogenetic tree generated from the partial polyprotein gene sequences grouped the BiH ArZ BA isolate within the third minor group together with the German isolates (S6 Fig; S1 File).

GFkV phylogenetic analyses, performed on the partial replicase gene sequences of four BiH isolates and 29 available reference isolates from 7 countries, clustered the isolates into two groups. The BiH isolates were within the major group, inside the subgroup with the Hungarian isolates (S7 Fig; S1 File). No recombinant events were detected in any of the BiH isolates of GLRaV-1, GFLV, ArMV and GFkV.

## Discussion

This study was conducted in various vineyards across the main grapevine cultivation areas in Bosnia and Herzegovina in order to investigate the sanitary status of two of the most important autochthonous cultivars, Žilavka and Blatina. The presence of grapevine viruses and a deteriorated sanitary status of vineyards in the country have emerged. All five major grapevine viruses (GLRaV-1, GLRaV-3, GFLV, GFkV and ArMV) have been detected, albeit with different incidences.

The dominant symptoms observed during the field survey were downward rolling with reddening (cv. Blatina) or yellowing (cv. Žilavka) of the leaves between the main green veins. These symptoms indicate the presence of grapevine leafroll-associated viruses (GLRaV-1 and -3), and in particular GLRaV-3, which resulted to be the most prevalent virus in both single and mixed infections. The symptoms here observed fit with those described by Delić et al. [61] on the same cultivars affected by GLD from one vineyard in BiH. The observed delayed growth, leaves with distortions and asymmetry, and shortening of the internodes, all of which are associated with GFDD, were similar to those previously described for GFLV involvement in this disease [62]. No GFkV symptoms were observed in plants that were found infected only by this virus, as expected due to the absence of symptoms of this virus on *V*. *vinifera* [19], while the ArMV symptoms detected in one grapevine plant in this study were similar to those described for GFDD. Noteworthy, the majority of the infected plants were asymptomatic and no relevant differences in virus incidence between the symptomatic and asymptomatic grapevines was recorded. This result requires further investigations since the absence of the symptoms may be associated with various factors, such as environmental conditions, virus strain, or mixed virus infections [26,63]. Despite the negative influence of GLD on yield and chemical composition of berries in symptomatic plants compared to asymptomatic plants, Alabi et al. [63] reported important variation of these features between these groups of the plants related to seasonal changes. In our preliminary observations, no evident differences in the overall yield could be observed for both cultivars between asymptomatic and symptomatic plants; it is possible that seasonal impact, but also an evolutionary development of these viruses in the Balkan region, or the relative accumulation of these viruses in the plants are involved in symptom induction. Future investigations will be devoted to quantify the virus accumulation and evaluate their localization in symptomatic and asymptomatic plants, together with a systematic analysis of the chemical composition of the berries and the organoleptic features of wines thereof.

The dominant virus found in our study was GLRaV-3, a result that is in agreement with previous studies in BiH on both cultivars [20,61,64,65]. Similar reports have also been published in neighbouring Balkan countries. Vončina and co-workers [66] reported that GLRaV-3 is the most frequent virus in Croatia, after analysing a panel of native cultivars (including cv. Žilavka) and found a similar level of incidence (79%) to that determined in this study, and to that (70%) reported by Kostadinovska and co-workers [67] for North Macedonia. The prevalence of GLRaV-3 among the major grapevine viruses has also been reported in Montenegro and Serbia, albeit with somewhat lower levels (55% and 31%, respectively) [68,69]. Finally, GLRaV-3 has been reported as the most frequent grapevine virus throughout the Mediterranean basin [16].

The high infection rate by GLRaV-3 found in our study could be explained by using infected plant material and the presence of potential GLD vectors in the vineyards, since GLRaV-3 has been reported to spread rapidly from infected to virus-free plants, reaching around 80% of infection in 8 years (10% of infection per year) [27,70]. In this study, the main hemipteran species observed in vineyards infected with GLRaV-1 and GLRaV-3 were *P*. *ficus* and *P*. *corni*, known vectors of GLRaV-3 and GLRaV-1 [53,54,71]. Recently, mealybugs and soft scale insects have frequently been reported in Herzegovinian vineyards [72,73]. The prevalence of GLRaV-3 and the presence of mealybugs and scales in infected vineyards require further studies in order to investigate the ability of *P*. *ficus* and *P*. *corni* to transmit GLRaV-1 and GLRaV-3, as already reported [14,27–30].

The relatively high incidence of GFLV observed in this study is possibly related to the use of infected propagation material, since *X*. *index* has not been found in our work, nor was it found in a previous extensive *Xiphinema* sp. study across BiH in grapevine cultivation areas [74]. Furthermore, some vineyards from our study were established in 2010 and showed a much higher infection rate than older ones planted in 1984 (53% vs. 8%), suggesting that infected propagation material was used in these new vineyards. It also seems that cv. Žilavka is more tolerant to GFLV infection (22% infection rate) than cv. Blatina (60%), since both cultivars were planted in vineyards at the same time, and cv. Blatina showed more infection by this virus in all GFLV affected vineyards. Similar findings were reported in a previous study on Herzegowian vineyards [64].

The finding of only a single plant infected with ArMV out of the 630 tested is not surprising, since its vector, *X*. *diversicaudatum*, has not been detected neither in this study nor in previous records pertaining to BiH [74]. The low ArMV incidence could be associated with the absence of the vector and the limited presence of the virus in vegetatively propagated grapevine material. The virus is also rarely present or even absent in neighboring countries [68,75,76], but when the vector is present [74], the incidence of ArMV might increase (> 20%) [66].

When GFkV was present in mixed infections with GLRaV-3 and GFLV in cv. Blatina and with GLRaV-3 in cv. Žilavka, a noticable reduction of the vigour of the plants, and subseqeuntly, decreased yield and quality was observed in infected plants. Such symptomatology was not observed either in the single GFkV infection, or in any other mixed infections. The present result deserves particular attention, since we mainly found GFkV in mixed infections (approx. 90% in both cvs.), and this virus (although symptompless in *V*. *vinifera*) influenced negatively the growth, yield and juice quality in *V*. *vinifera* cvs. Albana and Trebbiano Romagnolo when combined with other virus diseases such as vein necrosis and vein mosiac [77].

Phylogenetic analyses of the GLRaV-3 *CP* gene portion, including 35 BiH and forty-five reference GenBank isolates, clustered the isolates into nine already established groups [14,59,60]. The major group I included all the isolates from Bosnia and Herzegovina together with the reference isolates, while the remaining reference isolates were assigned to groups I-VII, IX and X. Group I was exclusive in BiH, as it is predominant in the rest of the world [78,79]. A minor subgroup within the group I contained the BiH isolates (four of which were recombinants), and it was clustered next to the another divergent subgroup with three Chinese isolates (JX088241, JX088165 and JX088185) [58], confirming the high genetic diversity of the virus isolates within this group. The explanation for the prevalence of GLRaV-3 I variants over the other groups is possibly related to a higher fitness of these variants or their more efficient transmission by vectors relative to the other variants [79]. Like the previous studies [25,78,80], we were unable to find any association of the phylogenetic group with the geographic origin or the grapevine cultivar. Although the genetic diversity of the GLRaV-3 BiH isolates of the *CP* region (0.010) was lower than that found in other similar studies (0.058 in Spain [78]; 0.063 in Portugal [79]), it is noteworthy that we considered only two cultivars, while previous reports were conducted on a higher number of cultivars. Nonetheless, the genetic diversity values obtained in this study, which suggest a negative or purifying selection, indicate that GLRaV-3 isolates have been present in the country since a long period of time, as reported in other countries with long-lasting grapevine tradition of cultivation.

The recombination of RNA viruses, through an exchange of genetic segments, has been associated with the selection of other genomic features that control gene expression, and with the alteration of vector transmission specificity [81]. In our study, we have found recombinant isolates in GLRaV-3, but not for the other studied grapevine viruses. The start and the end recombination break points found in the BiH isolates were between 180 and 330 nt, which are within the recombination junctions (142–531 nt) already reported for all the other recombinants in the *CP* gene of GLRaV-3 [58,82]. Although the recombinants here detected do not have the same start and end break points with the isolates reported by Turturo et al. [82] and Farooq et al. [58], it is interesting to note that in all three studies the recombination occurred between the isolates of groups I and II (apart from some recombinants between the groups I and III) [58]. Moreover, the recombination occurred at the 5’ portion of the *CP* gene, which presents the most variable gene part [24]. The occurrence of recombination in the most diverse part of *CP* could be related to a change in interaction between the viral CP and vector cellular receptors (preferences in the transmission pathways) or to a change in the level of expression of the *CP* and successive cascade reactions (selection of genomic features which controls a gene expression), both of which require further investigations [79,81].

Considering GLRaV-1, the phylogenetic analyses assigned four of the studied isolates into three groups. This may indicate a lack of geographical distribution, as already reported [25,83–85] and the impact of using infected propagation material in the spread of this virus within the country. Although one GFLV and ArMv isolate from BiH were characterized, the phylogenetic analyses clustered each of them within the phylogenetic groups containing the isolates from different countries, i.e. from various European countries for GFLV and from Germany for ArMV. Since no *Xiphinema* spp., the vectors of both viruses, have been detected in this and in previous studies in BiH [74], the possible route for their introduction in the country might again be the use of infected propagation material. As far as GFkV is concerned, all the BiH isolates grouped together in the same subgroup with a few virus isolates from Hungary, which could be related to an exchange of infected propagation material, since the vector for this virus is not known [36,86]. Based on these findings, if we exclude in the future the use of infected propagation material, further spread of GLRaV-1 and GLRaV-3 might be facilitated due to the presence of mixed infections and of the mealybugs and scale vectors that could act as potential vectors, while the risk of GFLV, ArMV and GFkV spread would be less envisaged, as no vectors are present in this area.

Overall, this is the first study on the genetic variability of GLRaV-3, the predominant virus in BiH. In spite of the limited number of virus isolates considered, our results show a variability of GLRaV-3, and the local virus isolates clustered together with isolates of different geographic origin. A higher number of clones for each isolate would be useful to investigate the presence of different variants within the same isolate, as already reported in some studies on GLRaV-1, GLRaV-3 and GFLV [22,82,85].

The phytopathological legislation in Bosnia and Herzegovina prescribes testing of grapevine planting material (both scions and rootstocks) for the presence of five viruses (GLRaV-1, GLRaV-3, GFLV, GFkV and ArMV), but their complete absence is not yet mandatory. This has led to a deterioration of the current grapevine sanitary status of autochthonous cultivars in the country, since a high infection rate of GLRaV-3 and GFLV, the difficulty of finding the virus-free mother plants, and the absence of certified planting material probably contribute to further spread the viruses, in particular to new vineyards. In order to prevent this from happening, it is of crucial importance to start producing ‘virus-tested’ and ‘virus-free’ propagation material at the national level, as well as introducing regular monitoring and adequate controls of mealybugs and scale insects and to avoid establishing new vineyards in close proximity to highly infected vineyards. Furthermore, the introduction of next-generation sequencing techniques would be highly recommended for the identification of all the foreign RNA and DNA sequences present in grapevine samples, enabling the detection of new viruses, of agronomically important viruses, and of latent viruses present at low titers. Full virome analyses might also contribute to modify the BiH phytopathological legislation and introduce new candidate target viruses in future surveys of grapevine propagation material [87,88]. All these measures will help to preserve the six century-old autochthonous cultivars used mainly for the production of ‘high-quality’ wines in this country.

In conclusion, our study has revealed a deteriorated sanitary status of the local grapevine industry in Bosnia and Herzegovina. The high genetic diversity among the BiH isolates and the presence of a mixed virus infection highlight the need for both stricter controls on the import of infected grapevine plants, in particular from neighboring countries, and further investigations on the sanitary status of grapevine plants in the presently unmonitored regions of Bosnia and Herzegovina.

## Supporting information

S1 Fig Agarose gel electrophoresis of DNA amplicons obtained by multiplex reverse transcription-polymerase chain reaction from naturally infected grapevine samples. Lanes 1, 2, 3, samples infected by GLRaV-3 and GFLV; lane 4, positive control for GLRaV-3 and GFLV; lane 5, sample infected by GFkV; lane 6, sample infected by GLRaV-1 and GFLV; lane 7, positive control for GLRaV-1; lanes 8, 9, 12, samples infected by GLRaV-3, GLRaV-1 and GFLV; lanes 10, 11, samples infected by GLRaV-3 and GLRaV-1; lane 13, positive control for GLRaV-3 and GLRaV-1; lane 14, sample infected by GLRaV-3; lane 15, positive control for GLRaV-1 and GFkV; lanes 16, 18, samples infected by GLRaV-1 and GFkV; lane 17, sample infected by GLRaV-1; lane 19, sample infected by GLRaV-3, GLRaV-1 and GFkV; lanes 20, 22, 23, samples infected by GLRaV-3; lane 24, positive control for GLRaV-3; lane 21, sample infected by ArMV; W, water control; H, healthy grapevine sample; L, 100 bp DNA ladder.
(TIF)

S2 Fig Graphical representations of pairwise percent identity of nucleotides of the sequenced coat protein gene between the GLRaV-3 isolates from Bosnia and Herzegovina using SDTv1.0 program. Each colored key represents a percentage to the identity score between two sequences.
(TIF)

S3 Fig Graphical representations of pairwise percent identity of nucleotides of the sequenced coat protein gene between the GLRaV-3 isolates from Bosnia and Herzegovina and reference isolates using SDTv1.0 program. Each colored key represents a percentage to the identity score between two sequences.
(TIF)

S4 Fig Phylogenetic relatedness of grapevine leafroll-associated virus 1 isolates obtained from partial coat protein sequences, as inferred from a maximum likelihood analysis. Four BiH isolates (in bold) and sixty reference isolates were included in the analysis. The accession number and the origin of each isolate are indicated. The bootstrap consensus tree was inferred from 1000 replicates.
(TIF)

S5 Fig Phylogenetic relatedness of grapevine fanleaf virus isolates obtained from partial coat protein sequences, as inferred from a maximum likelihood analysis. One BiH isolate (in bold) and fifty-five reference isolates were included in the analysis. The accession number and the origin of each isolate are indicated. The bootstrap consensus tree was inferred from 1000 replicates.
(TIF)

S6 Fig Phylogenetic relatedness of Arabis mosaic virus isolates obtained from partial polyprotein gene sequences, as inferred from a maximum likelihood analysis. One BiH isolate (in bold) and seventy-two reference isolates were included in the analysis. The accession number and the origin of each isolate are indicated. The bootstrap consensus tree was inferred from 1000 replicates.
(TIF)

S7 Fig Phylogenetic relatedness of grapevine fleck virus isolates obtained from partial replicase gene sequences, as inferred from a maximum likelihood analysis. Four BiH isolates (in bold) and twenty-nine reference isolates were included in the analysis. The accession number and the origin of each isolate are indicated. The bootstrap consensus tree was inferred from 1000 replicates.
(TIF)

S1 Table RT-PCR conditions and list of the primers used for virus specific detection and sequencing. (DOCX)

S2 Table Distribution of grapevine viruses by single and mixed infections in cv. Žilavka by DAS ELISA. (DOCX)

S3 Table Distribution of grapevine viruses by single and mixed infections in cv. Blatina by DAS ELISA. (DOCX)

S4 Table List of virus isolates from Bosnia and Herzegovina used in the study with their corresponding origin, grapevine cultivar and accession number. (DOCX)

S1 File List of the nucleotide sequences of the BiH grapevine virus isolates used in the phylogenetic and recombination analyses. (DOCX)
