# Supplementary figures and images for: Survey of five major grapevine viruses infecting Blatina and Žilavka cultivars in Bosnia and Herzegovina
Source: PLoS One. 2021 Jan 22;16(1):e0245959. doi: 10.1371/journal.pone.0245959 (PMC7822351; doi:10.1371/journal.pone.0245959)

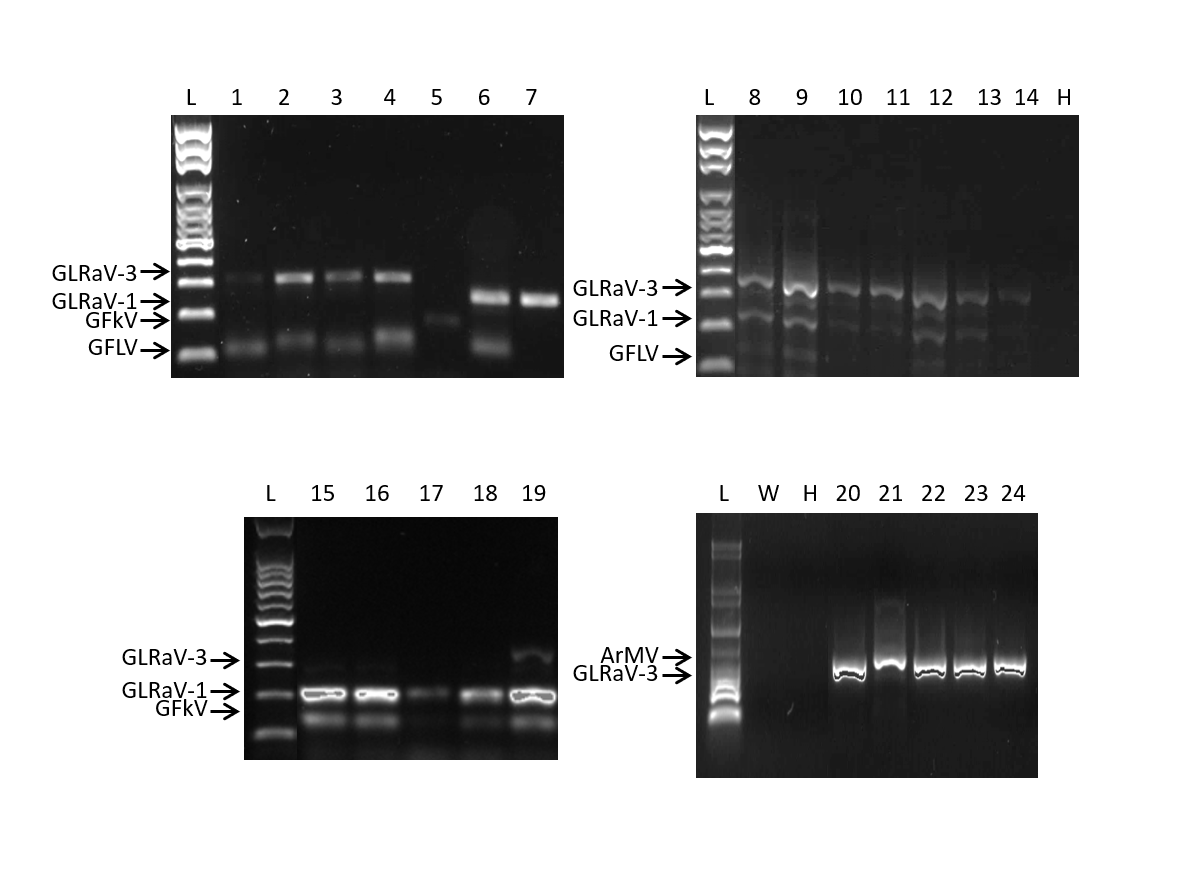

Supplement: S1 Fig — Lanes 1, 2, 3, samples infected by GLRaV-3 and GFLV; lane 4, positive control for GLRaV-3 and GFLV; lane 5, sample infected by GFkV; lane 6, sample infected by GLRaV-1 and GFLV; lane 7, positive control for GLRaV-1; lanes 8, 9, 12, samples infected by GLRaV-3, GLRaV-1 and GFLV; lanes 10, 11, samples infected by GLRaV-3 and GLRaV-1; lane 13, positive control for GLRaV-3 and GLRaV-1; lane 14, sample infected by GLRaV-3; lane 15, positive control for GLRaV-1 and GFkV; lanes 16, 18, samples infected by GLRaV-1 and GFkV; lane 17, sample infected by GLRaV-1; lane 19, sample infected by GLRaV-3, GLRaV-1 and GFkV; lanes 20, 22, 23, samples infected by GLRaV-3; lane 24, positive control for GLRaV-3; lane 21, sample infected by ArMV; W, water control; H, healthy grapevine sample; L, 100 bp DNA ladder. (TIF) [file pone.0245959.s001.tif]

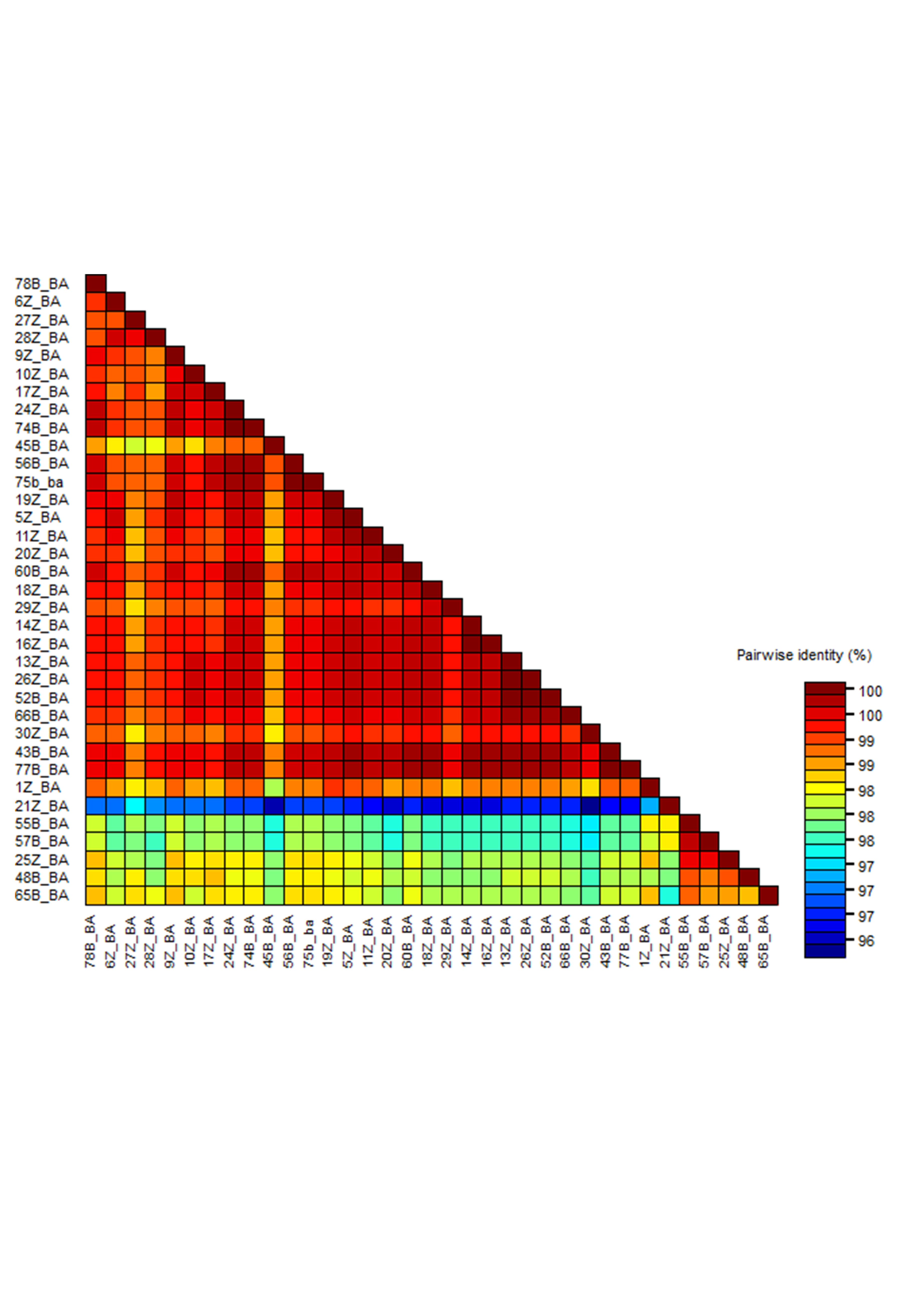

Supplement: S2 Fig — Each colored key represents a percentage to the identity score between two sequences. (TIF) [file pone.0245959.s002.tif]

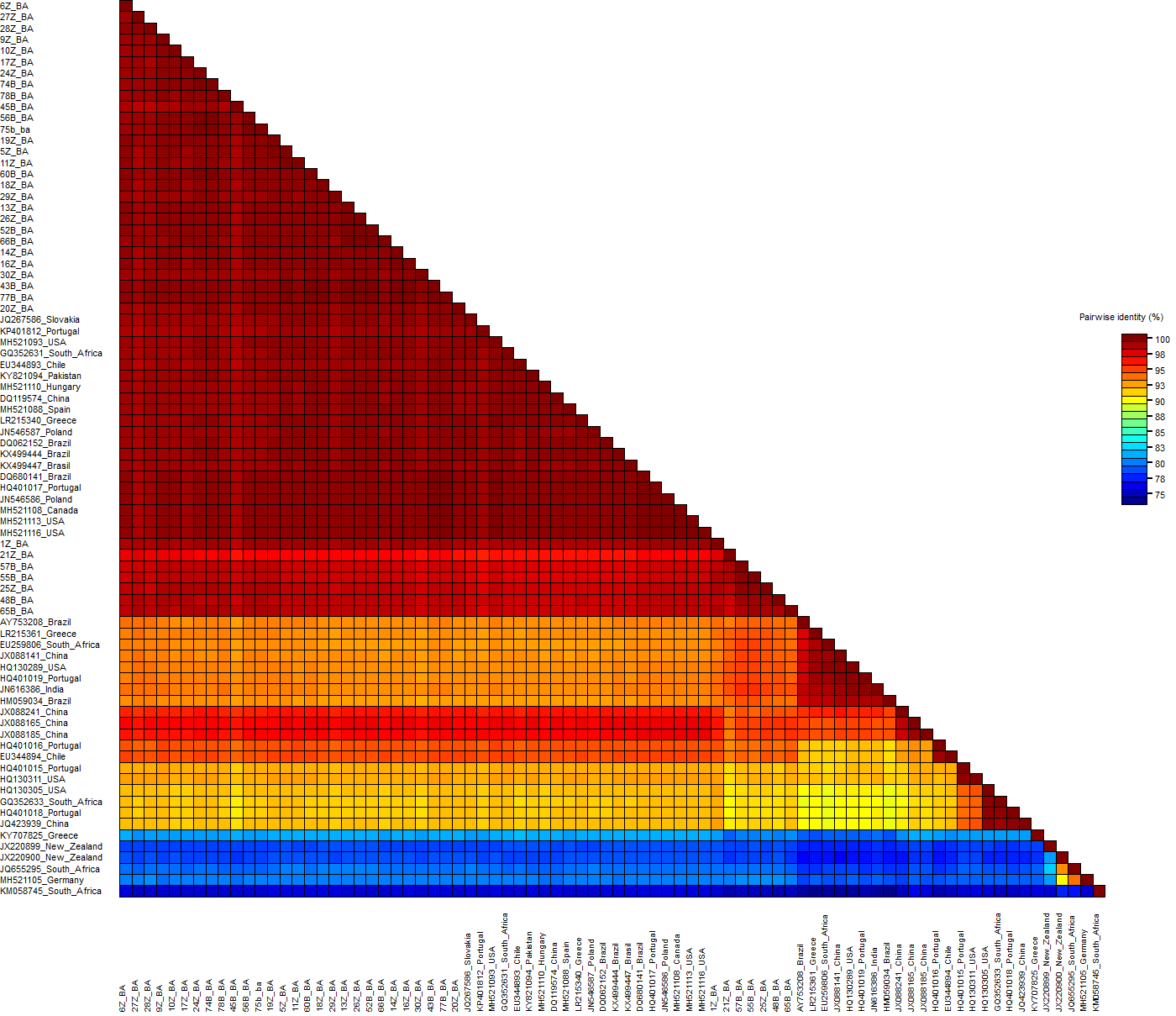

Supplement: S3 Fig — Each colored key represents a percentage to the identity score between two sequences. (TIF) [file pone.0245959.s003.tif]

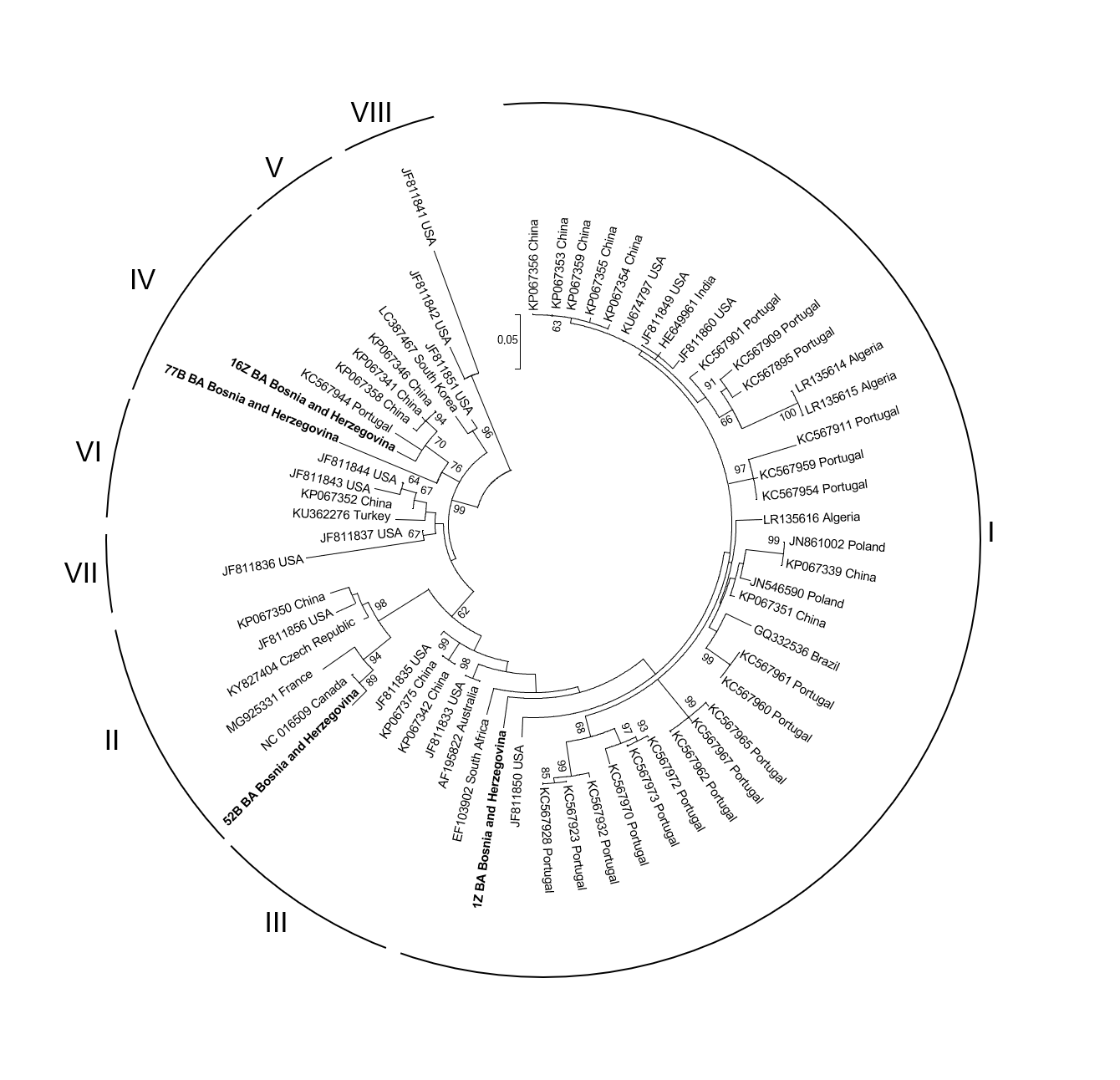

Supplement: S4 Fig — Four BiH isolates (in bold) and sixty reference isolates were included in the analysis. The accession number and the origin of each isolate are indicated. The bootstrap consensus tree was inferred from 1000 replicates. (TIF) [file pone.0245959.s004.tif]

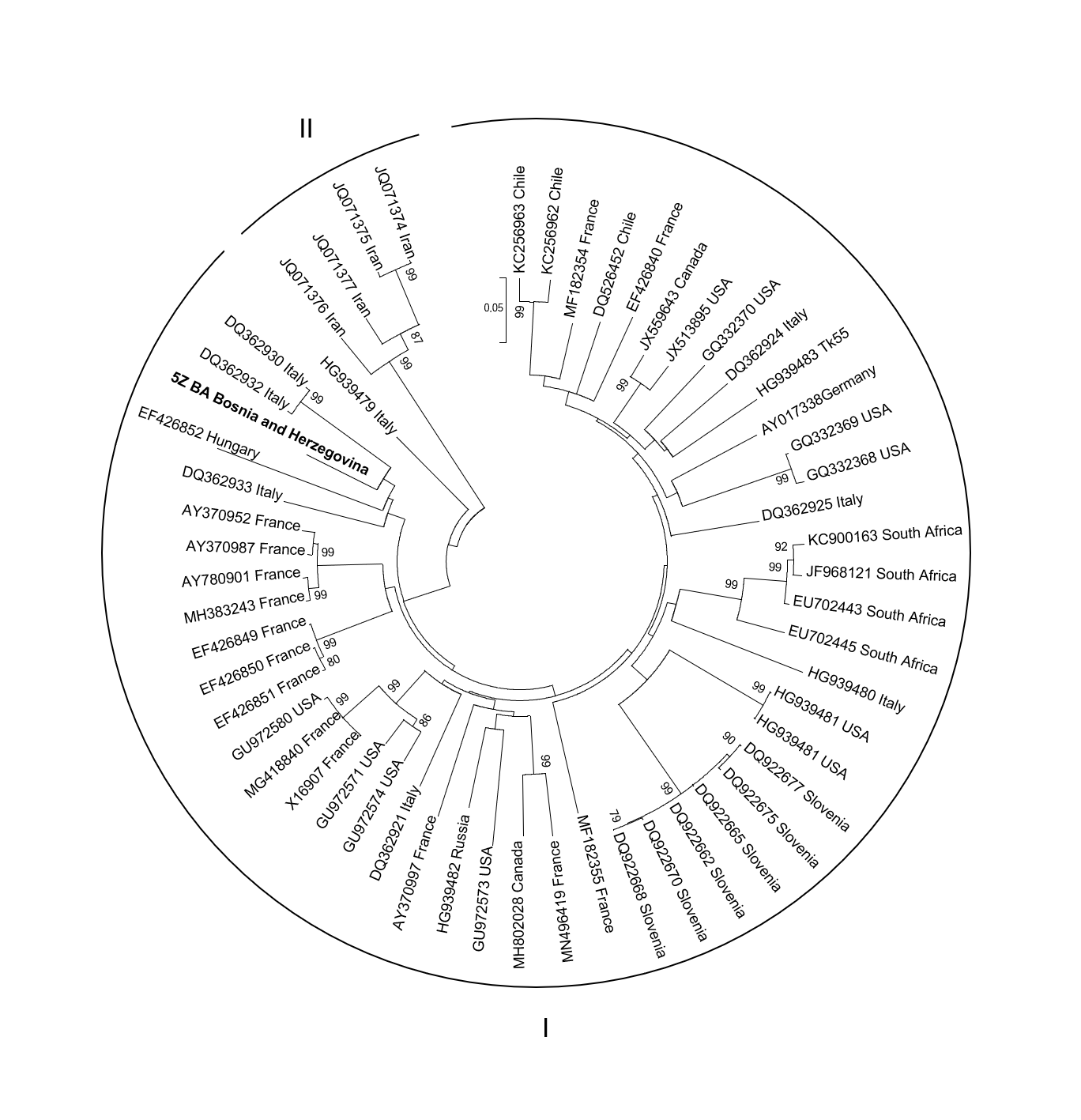

Supplement: S5 Fig — One BiH isolate (in bold) and fifty-five reference isolates were included in the analysis. The accession number and the origin of each isolate are indicated. The bootstrap consensus tree was inferred from 1000 replicates. (TIF) [file pone.0245959.s005.tif]

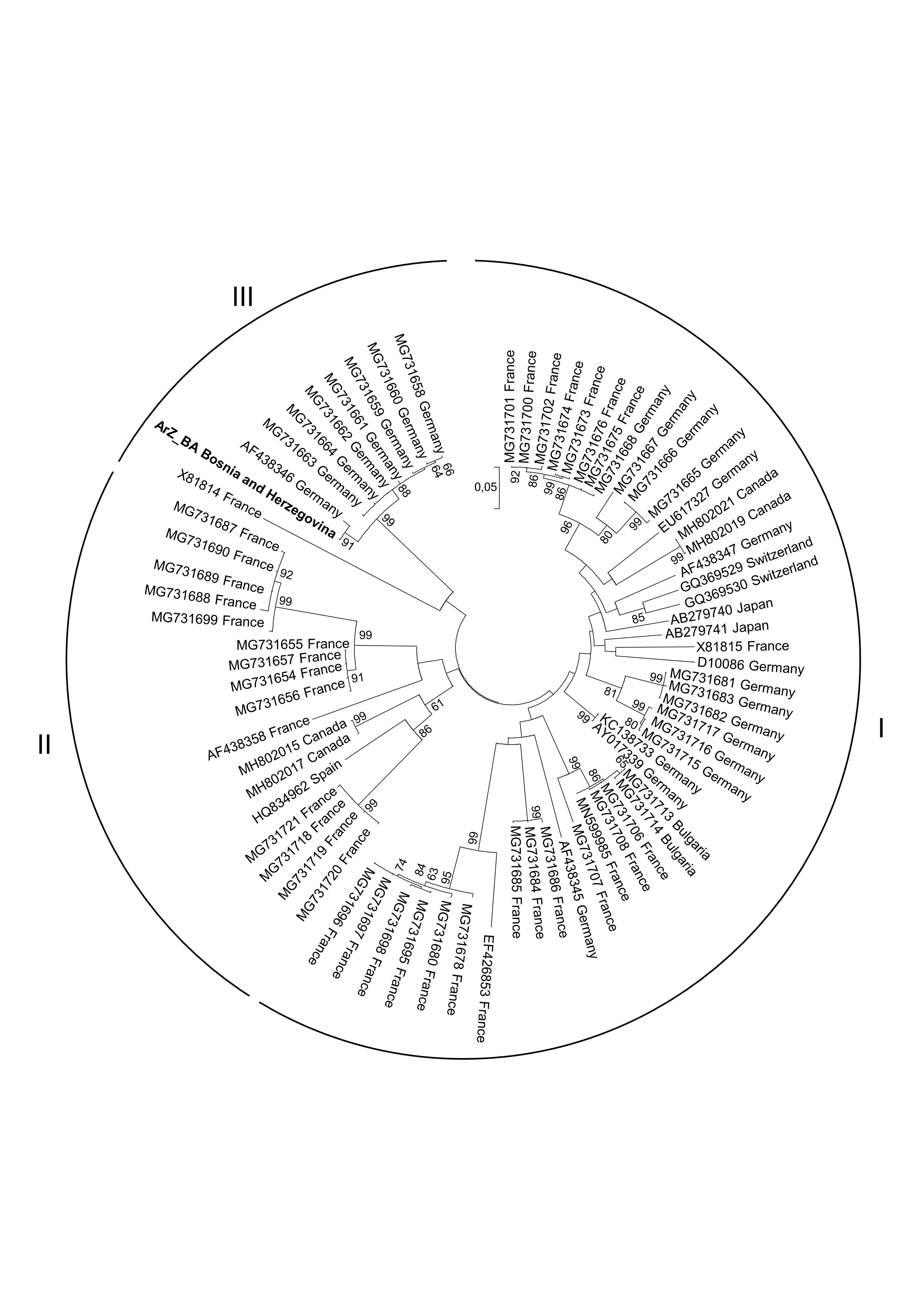

Supplement: S6 Fig — One BiH isolate (in bold) and seventy-two reference isolates were included in the analysis. The accession number and the origin of each isolate are indicated. The bootstrap consensus tree was inferred from 1000 replicates. (TIF) [file pone.0245959.s006.tif]

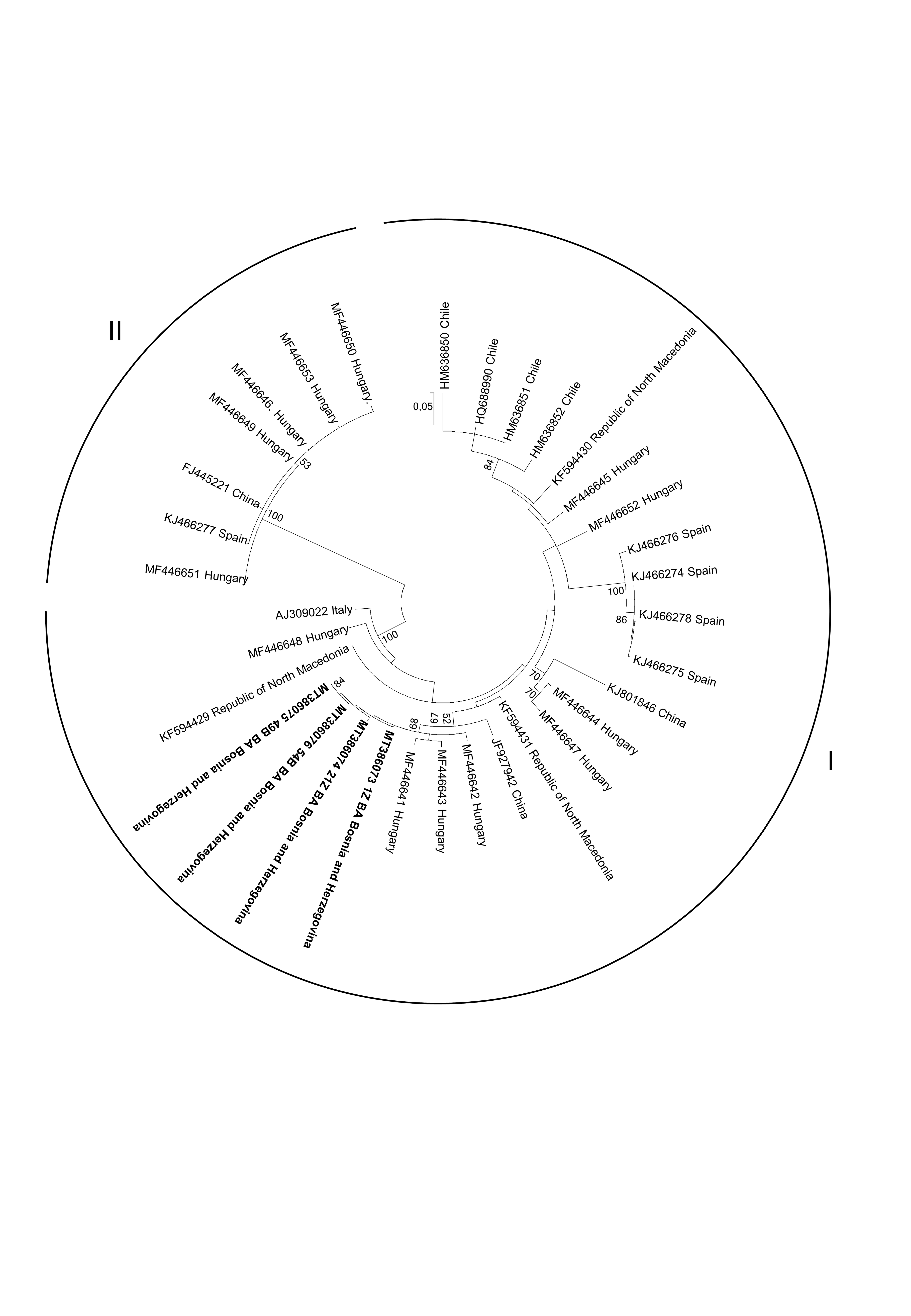

Supplement: S7 Fig — Four BiH isolates (in bold) and twenty-nine reference isolates were included in the analysis. The accession number and the origin of each isolate are indicated. The bootstrap consensus tree was inferred from 1000 replicates. (TIF) [file pone.0245959.s007.tif]
