# Supplementary material for: Survey of five major grapevine viruses infecting Blatina and Žilavka cultivars in Bosnia and Herzegovina
Source: PLoS One. 2021 Jan 22;16(1):e0245959. doi: 10.1371/journal.pone.0245959 (PMC7822351; doi:10.1371/journal.pone.0245959)
Supplement: S1 Table — (DOCX) [file pone.0245959.s008.docx]

**S1 Table.** RT-PCR conditions and list of the primers used for virus specific detection and sequencing.

| Virus | Gene | PCR conditions | Primer | Sequence (5’-3’) | Amplicon size (bp) | Reference |
| --- | --- | --- | --- | --- | --- | --- |
| GLRaV-3 | Coat protein | 47°C, 50 min; 95°C, 2min;  35 ×  (95°C, 1min; 48°C, 1min; 72°C, 1min); 72°C, 7min | LR3_8504V | ATGGCATTTGAACTGAAATT | 942 | Fajardo et al., 2007 |
|  |  |  | LR3_9445C | CTACTTCTTTTGCAATAGTT |  |  |
| GLRaV-1 | Coat protein | 47°C, 50 min; 94°C, 4min;  35 ×  (94°C, 30sec; 50°C, 1min; 72°C, 1min, 30 sec); 72°C, 7min | Forward | TCTTTACCAACCCCGAGATGAA | 232 | Gambino and Gribaudo, 2006 |
|  |  |  | Reverse | GTGTCTGGTGACGTGCTAAACG |  |  |
| GFLV | Coat protein | 47°C, 50 min; 94°C, 3min;  30 ×  (94°C, 30sec; 55°C, 60sec; 72°C, 1min, 30 sec); 72°C, 7min | EV00N1 | GACTATCTAGACACATATATACACTTGGGTCTTTTAA | 1573 | Vigne et al., 2004 |
|  |  |  | EV00N3 | ACTGTCTAGAGGATTRGCYGGYAGAGGAGT |  |  |
| ArMV | Polyprotein | 47°C, 50 min; 94°C, 4min;  35 ×  (94°C, 30sec; 54°C, 1min; 72°C, 1min, 30 sec); 72°C, 7min | ArMV 2BF | AGGGTCGCTTCTAGTACAGC | 962 | This study |
|  |  |  | ArMV 2BR | ATCCGAGGAAGAGCAACTCC |  |  |
