# Supplementary material for: Survey of five major grapevine viruses infecting Blatina and Žilavka cultivars in Bosnia and Herzegovina
Source: PLoS One. 2021 Jan 22;16(1):e0245959. doi: 10.1371/journal.pone.0245959 (PMC7822351; doi:10.1371/journal.pone.0245959)
Supplement: S2 Table — (DOCX) [file pone.0245959.s009.docx]

**S2 Table.** Distribution of grapevine viruses by single and mixed infections in cv. Žilavka by DAS ELISA.

| **Vineyards** | **Percentage**  **of samples**  **in relation to total number** | **Number of tested samples** | **Number of infected samples** | **Percentage (%)** | **GLRaV-1** | **GLRaV-3** | | | **GFLV** | **GFkV** | **ArMV** | **GLRaV-1**  **+**  **GLRaV-3** | **GLRaV-1**  **+**  **GFkV** | **GLRaV-3**  **+**  **GFLV** | **GLRaV-3**  **+**  **GFkV** | **GLRaV-1**  **+**  **GLRaV-3**  **+**  **GFLV** | **GLRaV-1**  **+**  **GLRaV-3**  **+**  **GFkV** | **GLRaV-3**  **+**  **GFLV**  **+**  **GFkV** |
| --- | --- | --- | --- | --- | --- | --- | --- | --- | --- | --- | --- | --- | --- | --- | --- | --- | --- | --- |
|  |  |  |  |  |  | | **Total (symptomatic/asymptomatic)** | | | | | | | | | | | |
| Višići, ČA | 6.12 | 17 | 17 | **100** | 0 | 6 (2/4) | | | 0 | 0 | 1 (1/0) | 1 (0/1) | 0 | 5 (1/4) | 3 (1/2) | 1 (0/1) | 0 | 0 |
| Blizanci, ČI | 7.55 | 21 | 12 | **57.14** | 0 | 8 (3/5) | | | 0 | 1 (0/1) | 0 | 1 (0/1) | 0 | 1 (0/1) | 1 (0/1) | 0 | 0 | 0 |
| Dugolaza-Ražovina, LJB | 15.83 | 44 | 40 | **90.91** | 2 (0/2) | 33 (10/23) | | | 1 (0/1) | 0 | 0 | 2 (1/1) | 0 | 0 | 2 (1/1) | 0 | 0 | 0 |
| Kosor, MO | 53.96 | 150 | 135 | **90** | 2 (1/1) | 75 (24/51) | | | 8 (2/6) | 2 (0/2) | 0 | 7 (2/5) | 4 (2/2) | 24 (4/20) | 3 (1/2) | 4 (1/3) | 5 (1/4) | 2 (1/1) |
| Plantaže-Otok, LJB | - | 0 | 0 | **-** | 0 | 0 | | | 0 | 0 | 0 | 0 | 0 | 0 | 0 | 0 | 0 | 0 |
| Sovići, GR | - | 0 | 0 | **-** | 0 | 0 | | | 0 | 0 | 0 | 0 | 0 | 0 | 0 | 0 | 0 | 0 |
| Buna Stup, MO | 6.12 | 17 | 13 | **76.47** | 1 (0/1) | 7 (2/5) | | | 1 (0/1) | 0 | 0 | 1 (0/1) | 0 | 3 (0/3) | 0 | 0 | 0 | 0 |
| Poprati, ST | 10.43 | 29 | 29 | **100** | 0 | 16 (5/11) | | | 0 | 0 | 0 | 3 (1/2) | 0 | 1 (0/1) | 3 (2/1) | 2 (0/2) | 3 (1/2) | 0 |
| **Total** | **100**  **-** | **278 -** | **246 (70/176)** | **88.49** | **5 (1/4)** | **145 (46/99)** | | | **10 (2/8)** | **3 (0/3)** | **1 (1/0)** | **15 (4/11)** | **4**  **(2/2)** | **34 (5/29)** | **12**  **(5/7)** | **7 (1/6)** | **8 (2/6)** | **2 (1/1)** |
| **Percentage (%)** |  |  | **88.49** |  | **2.03** | **58.94** | | **4.07** | | **1.22** | **0.41** | **6.10** | **1.63** | **13.82** | **4.88** | **2.85** | **3.25** | **0.81** |
