# Supplementary material for: Survey of five major grapevine viruses infecting Blatina and Žilavka cultivars in Bosnia and Herzegovina
Source: PLoS One. 2021 Jan 22;16(1):e0245959. doi: 10.1371/journal.pone.0245959 (PMC7822351; doi:10.1371/journal.pone.0245959)
Supplement: S3 Table — (DOCX) [file pone.0245959.s010.docx]

**S3 Table.** Distribution of grapevine viruses by single and mixed infections in cv. Blatina by DAS ELISA.

| **Vineyards** | **Percentage of samples in relation to total number** | **Number of tested samples** | **Number of infected samples** | **Percentage (%)** | **GLRaV-1** | **GLRaV-3** | | **GFLV** | **GFkV** | **ArMV** | **GLRaV-1**  **+**  **GLRaV-3** | **GLRaV-1**  **+**  **GFLV** | **GLRaV-3**  **+**  **GFLV** | **GLRaV-3**  **+**  **GFkV** | **GLRaV-1**  **+**  **GLRaV-3**  **+**  **GFLV** | **GLRaV-1**  **+**  **GLRaV-3**  **+**  **GFkV** | **GLRaV-3**  **+**  **GFLV**  **+**  **GFkV** |
| --- | --- | --- | --- | --- | --- | --- | --- | --- | --- | --- | --- | --- | --- | --- | --- | --- | --- |
|  |  |  |  |  | **Total (symptomatic/asymptomatic)** | | | | | | | | | | | | |
| Višići, ČA | 5.11 | 18 | 18 | **100.00** | 0 | 6 (1/5) | 1 (1/0) | | 0 | 0 | 2 (0/2) | 0 | 5 (2/3) | 0 | 3 (1/2) | 0 | 1 (0/1) |
| Blizanci, ČI | - | 0 | 0 | **-** | 0 | 0 | 0 | | 0 | 0 | 0 | 0 | 0 | 0 | 0 | 0 | 0 |
| Dugolaza-Ražovina, LJB | 14.20 | 50 | 49 | **98.00** | 0 | 19 (3/16) | 1 (0/1) | | 0 | 0 | 1 (0/1) | 0 | 9 (1/8) | 4 (1/3) | 0 | 1 (0/1) | 14 (3/11) |
| Kosor, MO | 46.31 | 163 | 124 | **76.07** | 6 (2/4) | 40 (6/34) | 13 (6/7) | | 3 (0/3) | 0 | 5 (2/3) | 2 (1/1) | 30 (4/26) | 12 (4/8) | 6 (2/4) | 3 (1/2) | 4 (1/3) |
| Plantaže-Otok, LJB | 14.77 | 52 | 51 | **98.08** | 1 (0/1) | 0 | 16 (5/11) | | 0 | 0 | 0 | 0 | 18 (3/15) | 0 | 4 (1/3) | 1 (0/1) | 11 (2/9) |
| Sovići, GR | 14.20 | 50 | 50 | **100.00** | 0 | 6 (2/4) | 16 (6/10) | | 1 (0/1) | 0 | 0 | 0 | 14 (4/10) | 4 (2/2) | 0 | 0 | 9 (3/6) |
| Buna Stup, MO | 5.40 | 19 | 15 | **78.95** | 2 (1/1) | 5 (1/4) | 2 (0/2) | | 1 (0/1) | 0 | 0 | 0 | 4 (1/3) | 1 (0/1) | 0 | 0 | 0 |
| Poprati, ST | - | 0 | 0 | **-** | 0 | 0 | 0 | | 0 | 0 | 0 | 0 | 0 | 0 | 0 | 0 | 0 |
| **Total** | **100 -** | **352 -** | **307**  **(73/234)** | **87.22** | **9**  **(3/6)** | **76**  **(13/63)** | **49 (18/31)** | | **5 (0/5)** | **0**  **-** | **8**  **(2/6)** | **2 (1/1)** | **80**  **(15/65)** | **21 (7/14)** | **13 (4/9)** | **5 (1/4)** | **39 (9/30)** |
| **Percentage (%)** |  |  | **87.22** |  | **2.93** | **24.76** | **15.96** | | **1.63** | **0.00** | **2.61** | **0.65** | **26.06** | **6.84** | **4.23** | **1.63** | **12.70** |
