# Supplementary material for: Survey of five major grapevine viruses infecting Blatina and Žilavka cultivars in Bosnia and Herzegovina
Source: PLoS One. 2021 Jan 22;16(1):e0245959. doi: 10.1371/journal.pone.0245959 (PMC7822351; doi:10.1371/journal.pone.0245959)
Supplement: S4 Table — (DOCX) [file pone.0245959.s011.docx]

**S4 Table.** List of virus isolates from Bosnia and Herzegovina used in the study with their corresponding origin, grapevine cultivar and accession number.

| **No** | **Isolate** | | **Origin** | **Cultivar** | **Accession No** | | | |  | **Virus infection by**  **ELISA/multiplex RT-PCR** |
| --- | --- | --- | --- | --- | --- | --- | --- | --- | --- | --- |
|  |  |  |  |  | **GLRaV-3** | **GLRaV-1** | **GFLV** | **GFkV** | **ArMV** |  |
| 1. | **ArZ_BA** | | Višići | Žilavka | - | - | - | - | MW413756 | ArMV |
| 2. | **1Z_BA** | | Višići | Žilavka | MT432352 | MK526895 | - | MT386073 | - | GLRaV-1, GLRaV-3, GFkV |
| 3. | 2Z_BA | | Višići | Žilavka | - | - | - | - | - | GLRaV-3 |
| 4. | 3Z_BA | | Blizanci | Žilavka | - | - | - | - | - | GLRaV-3 |
| 5. | 4Z_BA | | Blizanci | Žilavka | - | - | - | - | - | GLRaV-3, GFkV |
| 6. | **5Z_BA** | | Blizanci | Žilavka | MT432353 | - | MW147746 | - | - | GLRaV-3, GFkV, GFLV |
| 7. | **6Z_BA** | | Blizanci | Žilavka | MT432354 | - | - | - | - | GLRaV-3 |
| 8. | 7Z_BA | | Dugolaza/Ražovina | Žilavka | - | - | - | - | - | GLRaV-1, GLRaV-3, GFLV |
| 9. | 8Z_BA | | Dugolaza/Ražovina | Žilavka | - | - | - | - | - | GLRaV-3 |
| 10 | **9Z_BA** | | Dugolaza/Ražovina | Žilavka | MT432355 | - | - | - | - | GLRaV-3 |
| 11. | **10Z_BA** | | Dugolaza/Ražovina | Žilavka | MT432356 | - | - | - | - | GLRaV-3 |
| 12. | **11Z_BA** | | Dugolaza/Ražovina | Žilavka | MT432357 | - | - | - | - | GLRaV-3 |
| 13. | 12Z_BA | | Dugolaza/Ražovina | Žilavka | - | - | - | - | - | GLRaV-3, GFLV |
| 14. | **13Z_BA** | | Dugolaza/Ražovina | Žilavka | MT432358 | - | - | - | - | GLRaV-3 |
| 15. | **14Z_BA** | | Dugolaza/Ražovina | Žilavka | MT432359 | - | - | - | - | GLRaV-3 |
| 16. | 15Z_BA | | Dugolaza/Ražovina | Žilavka | - | - | - | - | - | GLRaV-3 |
| 17. | **16Z_BA** | | Kosor | Žilavka | MT432360 | MK526896 | - | - | - | GLRaV-1, GLRaV-3 |
| 18. | **17Z_BA** | | Kosor | Žilavka | MT432361 | - | - | - | - | GLRaV-1, GLRaV-3 |
| 19. | **18Z_BA** | | Kosor | Žilavka | MT432362 | - | - | - | - | GLRaV-3 |
| 20. | **19Z_BA** | | Kosor | Žilavka | MT432363 | - | - | - | - | GLRaV-3 |
| 21. | **20Z_BA** | | Kosor | Žilavka | MT432364 | - | - | - | - | GLRaV-3 |
| 22. | **21Z_BA** | | Kosor | Žilavka | MT432365 | - | - | MT386074 | - | GLRaV-3, GFkV |
| 23. | 22Z_BA | | Kosor | Žilavka | - | - | - | - | - | GLRaV-3 |
| 24. | 23Z_BA | | Kosor | Žilavka | - | - | - | - | - | GLRaV-1, GLRaV-3, GFLV |
| 25. | **24Z_BA** | | Kosor | Žilavka | MT432366 | - | - | - | - | GLRaV-3 |
| 26. | **25Z_BA** | | Kosor | Žilavka | MT432367 | - | - | - | - | GLRaV-3 |
| 27. | **26Z_BA** | | Kosor | Žilavka | MT432368 | - | - | - | - | GLRaV-3 |
| 28. | **27Z_BA** | | Kosor | Žilavka | MT432369 | - | - | - | - | GLRaV-1, GLRaV-3 |
| 29. | **28Z_BA** | | Kosor | Žilavka | MT432370 | - | - | - | - | GLRaV-3 |
| 30. | **29Z_BA** | | Kosor | Žilavka | MT432371 | - | - | - | - | GLRaV-3 |
| 31. | **30Z_BA** | | Kosor | Žilavka | MT432372 | - | - | - | - | GLRaV-3 |
| 32. | 31Z_BA | | Kosor | Žilavka | - | - | - | - | - | GLRaV-3 |
| 33. | 32Z_BA | | Kosor | Žilavka | - | - | - | - | - | GLRaV-3 |
| 34. | 33Z_BA | | Buna Stup | Žilavka | - | - | - | - | - | GLRaV-3 |
| 35. | 34Z_BA | | Buna Stup | Žilavka | - | - | - | - | - | GLRaV-3, GFkV |
| 36. | 35Z_BA | | Buna Stup | Žilavka | - | - | - | - | - | GLRaV-3, GFLV |
| 37. | 36Z_BA | | Poprati | Žilavka | - | - | - | - | - | GLRaV-3, GFkV |
| 38. | 37Z_BA | | Poprati | Žilavka | - | - | - | - | - | GLRaV-3, GFkV |
| 39. | 38Z_BA | | Poprati | Žilavka | - | - | - | - | - | GLRaV-3 |
| 40. | 39Z_BA | | Poprati | Žilavka | - | - | - | - | - | GLRaV-3 |
| 41 | 40Z_BA | | Poprati | Žilavka | - | - | - | - | - | GLRaV-3 |
| 42 | 41B_BA | | Višići | Blatina | - | - | - | - | - | GLRaV-3, GFkV |
| 43 | 42B_BA | | Višići | Blatina | - | - | - | - | - | GLRaV-3 |
| 44 | **43B_BA** | | Višići | Blatina | MT432373 | - | - | - | - | GLRaV-3 |
| 45 | 44B_BA | | Dugolaza/Ražovina | Blatina | - | - | - | - | - | GLRaV-3, GFLV |
| 46 | **45B_BA** | | Dugolaza/Ražovina | Blatina | MT432374 | - | - | - | - | GLRaV-3 |
| 47 | 46B_BA | | Dugolaza/Ražovina | Blatina | - | - | - | - | - | GLRaV-1, GLRaV-3 |
| 48 | 47B_BA | | Dugolaza/Ražovina | Blatina | - | - | - | - | - | GLRaV-3, GFkV |
| 49 | **48B_BA** | | Kosor | Blatina | MT432375 | - | - | - | - | GLRaV-3 |
| 50 | **49B_BA** | | Kosor | Blatina | - | - | - | MT386075 | - | GLRaV-3, GFkV |
| 51 | 50B_BA | | Kosor | Blatina | - | - | - | - | - | GLRaV-3, GFLV |
| 52 | 51B_BA | | Kosor | Blatina | - | - | - | - | - | GLRaV-3 |
| 53 | **52B_BA** | | Kosor | Blatina | MT432376 | MK526897 | - | - | - | GLRaV-1, GLRaV-3 |
| 54 | 53B_BA | | Kosor | Blatina | - | - | - | - | - | GLRaV-3 |
| 55 | **54B_BA** | | Kosor | Blatina | - | - | - | MT386076 | - | GLRaV-3, GFkV |
| 56 | **55B_BA** | | Kosor | Blatina | MT432377 | - | - | - | - | GLRaV-3 |
| 57 | **56B_BA** | | Kosor | Blatina | MT432378 | - | - | - | - | GLRaV-3 |
| 58 | **57B_BA** | | Kosor | Blatina | MT432379 | - | - | - | - | GLRaV-3 |
| 59 | 58B_BA | | Kosor | Blatina | - | - | - | - | - | GLRaV-3 |
| 60 | 59B_BA | | Kosor | Blatina | - | - | - | - | - | GLRaV-3, GFkV |
| 61 | **60B_BA** | | Kosor | Blatina | MT432380 | - | - | - | - | GLRaV-1, GLRaV-3 |
| 62 | | 61B_BA | Kosor | Blatina | - | - | - | - | - | GLRaV-3 |
| 63 | | 62B_BA | Kosor | Blatina | - | - | - | - | - | GLRaV-3, GFkV |
| 64 | | 63B_BA | Kosor | Blatina | - | - | - | - | - | GLRaV-3 |
| 65 | | 64B_BA | Kosor | Blatina |  | - | - | - | - | GLRaV-3 |
| 66 | | **65B_BA** | Plantaže/ | Blatina | MT432381 | - | - | - | - | GLRaV-3 |
| 67 | | **66B_BA** | Otok | Blatina | MT432382 | - | - | - | - | GLRaV-3 |
| 68 | | 67B_BA | Plantaže/ | Blatina | - | - | - | - | - | GFLV |
| 69 | | 68B_BA | Otok | Blatina | - | - | - | - | - | GLRaV-3, GFkV |
| 70 | | 69B_BA | Plantaže/ | Blatina | - | - | - | - | - | GLRaV-3, GFkV |
| 71 | | 70B_BA | Otok | Blatina | - | - | - | - | - | GLRaV-1, GLRaV-3 |
| 72 | | 71B_BA | Plantaže/ | Blatina | - | - | - | - | - | GLRaV-3, GFkV |
| 73 | | 72B_BA | Otok | Blatina | - | - | - | - | - | GLRaV-3 |
| 74 | | 73B_BA | Plantaže/ | Blatina | - | - | - | - | - | GLRaV-3 |
| 75 | | **74B_BA** | Otok | Blatina | MT432383 | - | - | - | - | GLRaV-3 |
| 76 | | **75B_BA** | Plantaže/ | Blatina | MT432384 | - | - | - | - | GLRaV-3, GFkV |
| 77 | | 76B_BA | Otok | Blatina | - | - | - | - | - | GLRaV-1, GLRaV-3, GFkV |
| 78 | | **77B_BA** | Plantaže/ | Blatina | MT432385 | MK526898 | - | - | - | GLRaV-1, GLRaV-3 |
| 79 | | **78B_BA** | Otok | Blatina | MT432386 | - | - | - | - | GLRaV-3, GFkV |
| 80 | | 79B_BA | Sovići | Blatina | - | - | - | - | - | GLRaV-3, GFkV |
| 81 | | 80B_BA | Sovići | Blatina | - | - | - | - | - | GLRaV-3 |

* The isolates in bold were sequenced in this study
