# Supplementary material for: Survey of five major grapevine viruses infecting Blatina and Žilavka cultivars in Bosnia and Herzegovina
Source: PLoS One. 2021 Jan 22;16(1):e0245959. doi: 10.1371/journal.pone.0245959 (PMC7822351; doi:10.1371/journal.pone.0245959)
Supplement: S1 File — (DOCX) [file pone.0245959.s012.docx]

>GLRaV-3 1Z_BA

ATGGCATTTGAACTGAAATTAGGGCAGATATATGAAGTCGTCCCCGAAAATAATTTGAGAGTTAGAGTAGGGGATGCGGCACAAGGAAAATTTAGTAAGGCGAGTTTCTTAAAGTACGTTAAGGACGGGACACAGGCGGAATTAACGGGAATCGCCGTAGTGCCCGAAAAATACGTATTCGCCACAGCAGCTTTAGCTACAGCGGCGCAGGAGCCACCTAAGCAGCCACCAGCGCAAGTGGTGGAACCACCAGAAGCTGATATAGGGGTAGTGCCGGAATCTGAGACTCTTACACCAAATAAGTTGGTTTTCGAGAAAGATCCAGACAAGTTCTTGAAGACTATGGGCAAGGGAATAGCTTTGGACTTGACGGGAGTTACCCATAAACCGAAAGTTATTAACGAGCCAGGGAAAGTATCAGTAGAGGTGGCAATGAAGATAAATGCCGCATTGATGGAGCTGTGTAAGAAGGTTATGGGCGCCGATGACGCAGCAACTAAGACAAAATTCTTCTTGTACGTGATGCAGATTGCTTGCACGTTCTTTACATCGTCTTCGACGGAGTTCAAAGAGTTTGACTACATAGAAACCGATGATGGAAAGAAGATATATGCGGTGTGGGTATATGATTGCATTAAACAAGCTGCTGCTTCGACGGGTTATGAAAACCCGGTAAGGCAGTATCTAGCGTACTTCACACCAACCTTCATCACGGCGACCCTGAATGGTAAACTAGTGATGAACGAGAAGGTTATGGCACAGCATGGAGTACCACCGAAATTCTTTCCGTACACGATAGACTGCGTTCGTCCGACGTACGATCTGTTCAACAACGACGCAATACTAGCATGGAATTTAGCTAGACAGCAGGCGTTTAGAAACAAGACGGTAACGGCCGATAACACCTTACACAACGTCTTCCAACTATTGCAAAAGAAGTAG

> GLRaV-3 5Z_BA

ATGGCATTTGAACTGAAATTAGGGCAGATATATGAAGTCGTCCCCGAAAATAATTTGAGAGTTAGAGTAGGGGATGCGGCACAAGGAAAATTTAGTAAGGCGAGTTTCTTAAAGTACGTTAAGGACGGGACACAGGCGGAATTAACGGGAATCGCCGTAGTGCCCGAAAAATACGTATTCGCCACAGCAGCTTTGGCTACAGCGGCGCAGGAGCCACCTAGGCAGCCACCAGCGCAAGTGGTGGAACCACCGGAAACCGATATAGGGGTAGTGCCGGAATCTGAGACTCTCACACCAAATAAGTTGGTTTTCGAGAAAGATCCAGACAAGTTCTTGAAGACTATGGGCAAGGGAATAGCTTTGGACTTGACGGGAGTTACCCACAAACCGAAAGTTATTAACGAGCCAGGGAAAGTATCAGTAGAGGTGGCAATGAAGATAAATGCCGCATTGATGGAGCTGTGTAAGAAGGTTATGGGCGCCGATGACGCAGCAACTAAGACAAAATTCTTCTTGTACGTGATGCAGATTGCTTGCACGTTCTTTACATCGTCTTCGACGGAGTTCAAAGAGTTTGACTACATAGAAACCGATGATGGAAAGAAGATATATGCGGTGTGGGTATATGATTGCATTAAACAAGCTGCTGCTTCGACGGGTTATGAAAACCCGGTAAGGCAGTATCTAGCGTACTTCACACCAACCTTCATCACGGCGACCCTGAATGGTAAACTAGTGATGAACGAGAAGGTTATGGCACAGCATGGAGTACCACCGAAATTCTTTCCGTACACGATAGACTGCGTTCGTCCGACGTACGATCTGTTCAACAACGACGCAATACTAGCATGGAATTTAGCTAGACAGCAGGCGTTTAGAAACAAGACGGTAACGGCCGATAACACCTTACACAACGTCTTCCAACTATTGCAAAAGAAGTAG

>GLRaV-3 6Z_BA

ATGGCATTTGAACTGAAATTAGGGCAGATATATGAAGTCGTCCCCGAAAATAATTTGAGAGTTAGAGTAGGGGATGCGGCACAAGGAAAATTTAGTAAGGCGAGTTTCTTAAAGTACGTTAAGGACGGGACACAGGCGGAATTAACGGGAATCGCCGTAGTGCCCGAAAAATACGTATTCGCCACAGCAGCTTTGGCTACAGCGGCGCAGGAGCCACCTAGGCAGCCACCAACGCAAGTGGTGGAACCACCGGAAACCGATATAGGGGTAGTGCCGGAATCTGAGACTCTCACACCAAATAAGTTGGTTTTCGAGAAAGATCCAGACAAGTTCTTAAAGACTATGGGCAAGGGAATAGCTTTGGACTTAACGGGAGTTACCCACAAACCGAAAGTTATTAACGAGCCAGGGAAAGTATCAGTAGAGGTGGCAATGAAGATAAATGCCGCATTGATGGAGCTGTGTAAGAAGGTTATGGGCGCCGATGACGCAGCAACTAAGACAAAATTCTTCTTGTACGTGATGCAGATTGCTTGCACGTTCTTTACATCGTCTTCGACGGAGTTCAAAGAGTTTGACTACATAGAAACCGATGATGGAAAGAAGATATATGCGGTGTGGGTATATGATTGCATTAAACAAGCTGCTGCTTCGACGGGTTATGAAAACCCGGTAAGGCAGTATCTAGCGTACTTCACACCAACCTTCATCACGGCGACCCTGAATGGTAAACTAGTGATGAACGAGAAGGTTATGGCACAGCATGGAGTACCACCGAAATTCTTTCCGTACACGATAGACTGCGTTCGTCCGACGTACGATCTGTTCAACAACGACGCAATACTAGCATGGAATTTAGCTAGACAGCAGGCGTTTAGAAACAAGACGGTAACGGCCGATAACACCTTACACAACGTCTTCCAACTATTGCAAAAGAAGTAG

>GLRaV-3 9Z_BA

ATGGCATTTGAACTGAAATTAGGGCAGATATATGAAGTCGTCCCCGAAAATAATTTGAGAGTTAGAGTAGGGGATGCGGCACAAGGAAAATTTAGTAAGGCGAGTTTCTTAAAGTACGTTAAGGACGGGACACAGGCGGAATTAACGGGAATCGCCGTAGTGCCCGAAAAATACGTATTCGCCACAGCAGCTTTGGCTACAGCGGCGCAGGAGCCACCTAAGCAGCCAACAGCGCAAGTGGTGGAACCACCGGAAACCGATATAGGGGTGGTGCCGGAATCTGAGACTCTCACACCAAATAAGTTGGTTTTCGAGAAAGATCCAGACAAGTTCTTGAAGACTATGGGCAAGGGAATAGCTTTGGACTTGACGGGAGTTACCCACAAACCGAAAGTTATTAACGAGCCAGGGAAAGTATCAGTAGAGGTGGCAATGAAGATAAATGCCGCATTGATGGAGCTGTGTAAGAAGGTTATGGGCGCCGATGACGCAGCAACTAAGACAAAATTCTTCTTGTACGTGATGCAGATTGCTTGCACGTTCTTTACATCGTCTTCGACGGAGTTCAAAGAGTTTGACTACATAGAAACCGATGATGGAAAGAAGATATATGCGGTGTGGGTATATGATTGCATTAAACAAGCTGCTGCTTCGACGGGTTATGAAAACCCGGTAAGGCAGTATCTAGCGTACTTCACACCAACCTTCATCACGGCGACCCTGAATGGTAAACTAGTGATGAACGAGAAGGTTATGGCACAGCATGGAGTACCACCGAAATTCTTTCCGTACACGATAGACTGCGTTCGTCCGACGTACGATCTGTTCAACAACGACGCAATACTAGCATGGAATTTAGCTAGACAGCAGGCGTTTAGAAACAAGACGGTAACGGCCGATAACACCTTACACAACGTCTTCCAACTATTGCAAAAGAAGTAG

>GLRaV-3 10Z_BA

ATGGCATTTGAACTGAAATTAGGGCAGATATATGAAGTCGTCCCCGAGAATAATTTGAGAGTTAGAGTAGGGGATGCGGCACAAGGAAAATTTAGTAAGGCGAGTTTCTTAAAGTACGTTAAGGACGGGACACAGGCGGAATTAACGGGAATCGCCGTAGTGCCCGAAAAATACGTATTCGCCACAGCAGCTTTGGCTACAGCGGCGCAGGAGCCACCTAAGCAGCCAACAGCGCAAGTGGTGGAACCACAGGAAACCGATATAGGGGTAGTGCCGGAATCTGAGACTCTCACACCAAATAAGTTGGTTTTCGAGAAAGATCCAGACAAGTTCTTGAAGACTATGGGTAAGGGAATAGCTTTGGACTTGACGGGAGTTACCCACAAACCGAAAGTTATTAACGAGCCAGGGAAAGTATCAGTAGAGGTGGCAATGAAGATAAATGCCGCATTGATGGAGCTGTGTAAGAAGGTTATGGGCGCCGATGACGCAGCAACTAAGACAAAATTCTTCTTGTACGTGATGCAGATTGCTTGCACGTTCTTTACATCGTCTTCGACGGAGTTCAAAGAGTTTGACTACATAGAAACCGATGATGGAAAGAAGATATATGCGGTGTGGGTATATGATTGCATTAAACAAGCTGCTGCTTCGACGGGTTATGAAAACCCGGTAAGGCAGTATCTAGCGTACTTCACACCAACCTTCATCACGGCGACCCTGAATGGTAAACTAGTGATGAACGAGAAGGTTATGGCACAGCATGGAGTACCACCGAAATTCTTTCCGTACACGATAGACTGCGTTCGTCCGACGTACGATCTGTTCAACAACGACGCAATACTAGCATGGAATTTAGCTAGACAGCAGGCGTTTAGAAACAAGACGGTAACGGCCGATAACACCTTACACAACGTCTTCCAACTATTGCAAAAGAAGTAG

>GLRaV-3 11Z_BA

ATGGCATTTGAACTGAAATTAGGGCAGATATATGAAGTCGTCCCCGAAAATAATTTGAGAGTTAGAGTAGGGGATGCGGCACAAGGAAAATTTAGTAAGGCGAGTTTCTTAAAGTACGTTAAGGACGGGACACAGGCGGAATTAACGGGAATCGCCGTAGTGCCCGAAAAATACGTATTCGCCACAGCAGCTTTGGCTACAGCGGCGCAGGAGCCACCTAGGCAGCCACCAGCGCAAGTGGTGGAACCACCGGAAACCGATATAGGGGTAGTGCCGGAATCTGAGACTCTCACACCAAATAAGTTGGTTTTCGAGAAAGATCCAGACAAGTTCTTGAAGACTATGGGCAAGGGAATAGCTTTGGACTTGACGGGAGTTACCCACAAACCGAAAGTTATTAACGAGCCAGGGAAAGTATCAGTAGAGGTGGCAATGAAGATAAATGCCGCATTGATGGAGCTGTGTAAGAAGGTTATGGGCGCCGATGACGCAGCAACTAAGACAAAATTCTTCTTGTACGTGATGCAGATTGCTTGCACGTTCTTTACATCGTCTTCGACGGAGTTCAAAGAGTTTGACTACATAGAAACCGATGATGGAAAGAAGATATATGCGGTGTGGGTATATGATTGCATTAAACAAGCTGCTGCTTCGACGGGTTATGAAAACCCGGTAAGGCAGTATCTAGCGTACTTCACACCAACCTTCATCACGGCGACCCTGAATGGTAAACTAGTGATGAACGAGAAGGTTATGGCACAGCATGGAGTACCACCGAAATTCTTTCCGTACACGATAGACTGCGTTCGTCCGACGTACGATCTGTTCAACAACGACGCAATACTAGCATGGAATTTAGCTAGACAGCAGGCGTTTAGAAACAAGACGGTAACGGCCGATAACACCTTACATAACGTCTTCCAACTATTGCAAAAGAAGTAG

>GLRaV-3 13Z_BA

ATGGCATTTGAACTGAAATTAGGGCAGATATATGAAGTCGTCCCCGAGAATAATTTGAGAGTTAGAGTAGGGGATGCGGCACAAGGAAAATTTAGTAAGGCGAGTTTCTTAAAGTACGTTAAGGACGGGACACAGGCGGAATTAACGGGAATCGCCGTAGTGCCCGAAAAATACGTATTCGCCACAGCAGCTTTGGCTACAGCGGCGCAGGAGCCACCTAGGCAGCCACCAGCGCAAGTGGTGGAACCACAGGAAACCGATATAGGGGTAGTGCCGGAATCTGAGACTCTCACACCAAATAAGTTGGTTTTCGAGAAAGATCCAGACAAGTTCTTGAAGACTATGGGCAAGGGAATAGCTTTGGACTTGACGGGAGTTACCCACAAACCGAAAGTTATTAACGAGCCAGGGAAAGTATCAGTAGAGGTGGCAATGAAGATAAATGCCGCATTGATGGAGCTGTGTAAGAAGGTTATGGGCGCCGATGACGCAGCAACTAAGACAAAATTCTTCTTGTACGTGATGCAGATTGCTTGCACGTTCTTTACATCGTCTTCGACGGAGTTCAAAGAGTTTGACTACATAGAAACCGATGATGGAAAGAAGATATATGCGGTGTGGGTATATGATTGCATTAAACAAGCTGCTGCTTCGACGGGTTATGAAAACCCGGTAAGGCAGTATCTAGCGTACTTCACACCAACCTTCATCACGGCGACCCTGAATGGTAAACTAGTGATGAACGAGAAGGTTATGGCACAGCATGGAGTACCACCGAAATTCTTTCCGTACACGATAGACTGCGTTCGTCCGACGTACGATCTGTTCAACAACGACGCAATACTAGCATGGAATTTAGCTAGACAGCAGGCGTTTAGAAACAAGACGGTAACGGCCGATAACACCTTACACAACGTCTTCCAACTATTGCAAAAGAAGTAG

>GLRaV-3 14Z_BA

ATGGCATTTGAACTGAAATTAGGGCAGATATATGAAGTCGTCCCCGAAAATAATTTGAGAGTTAGAGTAGGGGATGCGGCACAAGGAAAATTTAGTAAGGCGAGTTTCTTAAAGTACGTTAAGGACGGGACACAGGCGGAATTAACGGGAATCGCCGTAGTGCCCGAAAAATACGTATTCGCCACAGCAGCTTTGGCTACAGCGGCGCAGGAGCCAACTAGGCAGCCACCAGCGCAAGTGGTGGAACCACAGGAAACCGATATAGGGGTAGTGCCGGAATCTGAGACTCTCACACCAAATAAGTTGGTTTTCGAGAAAGATCCAGACAAGTTCTTGAAGACTATGGGCAAGGGAATAGCTTTGGACTTGACGGGAGTTACCCACAAACCGAAAGTTATTAACGAGCCAGGGAAAGTATCAGTAGAGGTGGCAATGAAGATAAATGCCGCATTGATGGAGCTGTGTAAGAAGGTTATGGGCGCCGATGACGCAGCAACTAAGACAAAATTCTTCTTGTACGTGATGCAGATTGCTTGCACGTTCTTTACATCGTCTTCGACGGAGTTCAAAGAGTTTGACTACATAGAAACCGATGATGGAAAGAAGATATATGCGGTGTGGGTATATGATTGCATTAAACAAGCTGCTGCTTCGACGGGTTATGAAAACCCGGTAAGGCAGTATCTAGCGTACTTCACACCAACCTTCATCACGGCGACCCTGAATGGTAAACTAGTGATGAACGAGAAGGTTATGGCACAGCATGGAGTACCACCGAAATTCTTTCCGTACACGATAGACTGCGTTCGTCCGACGTACGATCTGTTCAACAACGACGCAATACTAGCATGGAATTTAGCTAGACAGCAGGCGTTTAGAAACAAGACGGTAACGGCCGATAACACCTTACACAACGTCTTCCAACTATTGCAAAAGAAGTAG

>GLRaV-3 16Z_BA

ATGGCATTTGAACTGAAATTAGGGCAGATATATGAAGTCGTCCCCGAAAATAATTTGAGAGTTAGAGTAGGGGATGCGGCACAAGGAAAATTTAGTAAGGCGAGTTTCTTAAAGTACGTTAAGGACGGGACACAGGCGGAATTAACGGGAATCGCCGTAGTGCCCGAAAAATACGTATTCGCCACAGCAGCTTTGGCTACAGCGGCGCAGGAGCCAACTAGGCAGCCACCAGCGCAAGTGGTGGAACCACAGGAAACCGATATAGGGGTAGTGCCGGAATCTGAGACTCTCACACCAAATAAGTTGGTTTTCGAGAAAGATCCAGACAAGTTCTTGAAGACTATGGGCAAGGGAATAGCTTTGGACTTGACGGGAGTTACCCACAAACCGAAAGTTATTAACGAGCCAGGGAAAGTATCAGTAGAGGTGGCAATGAAGATAAATGCCGCATTGATGGAGCTGTGTAAGAAGGTTATGGGCGCCGATGACGCAGCAACTAAGACAAAATTCTTCTTGTACGTGATGCAGATTGCTTGCACGTTCTTTACATCGTCTTCGACGGAGTTCAAAGAGTTTGACTACATAGAAACCGATGATGGAAAGAAGATATATGCGGTGTGGGTATATGATTGCATTAAACAAGCTGCTGCTTCGACGGGTTATGAAAACCCGGTAAGGCAGTATCTAGCGTACTTCACACCAACCTTCATCACGGCGACCCTGAATGGTAAACTAGTGATGAACGAGAAGGTTATGGCACAGCATGGAGTACCACCGAAATTCTTTCCGTACACGATAGACTGCGTTCGTCCGACGTACGATCTGTTCAACAACGACGCAATACTAGCATGGAATTTAGCTAGACAGCAGGCGTTTAGAAACAAGACGGTAACGGCCGATAACACCTTACACAACGTCTTCCAACTATTGCAAAAGAAGTAG

>GLRaV-3 17Z_BA

ATGGCATTTGAACTGAAATTAGGGCAGATATATGAAGTCGTCCCCGAGAATAATTTGAGAGTTAGAGTAGGGGATGCGGCACAAGGAAAATTTAGTAAGGCGAGTTTCTTAAAGTACGTTAAGGACGGGACACAGGCGGAATTAACGGGAATCGCCGTAGTGCCCGAAAAATACGTATTCGCCACAGCAGCTTTGGCTACAGCGGCGCAGGAGCCACCTAAGCAGCCGACAGCGCAAGTGGTGGAACCACAGGAAACCGATATAGGGGTGGTGCCGGAATCTGAGACTCTCACACCAAATAAGTTGGTTTTCGAGAAAGATCCAGACAAGTTCTTGAAGACTATGGGCAAGGGAATAGCTTTGGACTTGACGGGAGTTACCCACAAACCGAAAGTTATTAACGAGCCAGGGAAAGTATCAGTAGAGGTGGCAATGAAGATAAATGCCGCATTGATGGAGCTGTGTAAGAAGGTTATGGGCGCCGATGACGCAGCAACTAAGACAAAATTCTTCTTGTACGTGATGCAGATTGCTTGCACGTTCTTTACATCGTCTTCGACGGAGTTCAAAGAGTTTGACTACATAGAAACCGATGATGGAAAGAAGATATATGCGGTGTGGGTATATGATTGCATTAAACAAGCTGCTGCTTCGACGGGTTATGAAAACCCGGTAAGGCAGTATCTAGCGTACTTCACACCAACCTTCATCACGGCGACCCTGAATGGTAAACTAGTGATGAACGAGAAGGTTATGGCACAGCATGGAGTACCACCGAAATTCTTTCCGTACACGATAGACTGCGTTCGTCCGACGTACGATCTGTTCAACAACGACGCAATACTAGCATGGAATTTAGCTAGACAGCAGGCGTTTAGAAACAAGACGGTAACGGCCGATAACACCTTACACAACGTCTTCCAACTATTGCAAAAGAAGTAG

>GLRaV-3 18Z_BA

ATGGCATTTGAACTGAAATTAGGGCAGATATATGAAGTCGTCCCCGAAAATAATTTGAGAGTTAGAGTAGGGGATGCGGCACAAGGAAAATTTAGTAAGGCGAGTTTCTTAAAGTACGTTAAGGACGGGACACAGGCGGAATTAACGGGAATCGCCGTAGTGCCCGAAAAATACGTATTCGCTACAGCAGCTTTGGCTACAGCGGCGCAGGAGCCACCTAGGCAGCCACCAGCGCAAGTGGTGGAACCACAGGAAACCGATATAGGGGTAGTGCCGGAATCTGAGACTCTCACACCAAATAAGTTGGTTTTCGAGAAAGATCCAGACAAGTTCTTGAAGACTATGGGCAAGGGAATAGCTTTGGACTTGACGGGAGTTACCCACAAACCGAAAGTTATTAACGAGCCAGGGAAAGTATCAGTAGAGGTGGCAATGAAGATAAATGCCGCATTGATGGAGCTGTGTAAGAAGGTTATGGGCGCCGATGACGCAGCAACTAAGACAAAATTCTTCTTGTACGTGATGCAGATTGCTTGCACGTTCTTTACATCGTCTTCGACGGAGTTCAAAGAGTTTGACTACATAGAAACCGATGATGGAAAGAAGATATATGCGGTGTGGGTATATGATTGCATTAAACAAGCTGCTGCTTCGACGGGTTATGAAAACCCGGTAAGGCAGTATCTAGCGTACTTCACACCAACCTTCATCACGGCGACCCTGAATGGTAAACTAGTGATGAACGAGAAGGTTATGGCACAGCATGGAGTACCACCGAAATTCTTTCCGTACACGATAGACTGCGTTCGTCCGACGTACGATCTGTTCAACAACGACGCAATACTAGCATGGAATTTAGCTAGACAGCAGGCGTTTAGAAACAAGACGGTAACGGCCGATAACACCTTACACAACGTCTTCCAACTATTGCAAAAGAAGTAG

>GLRaV-3 19Z_BA

ATGGCATTTGAACTGAAATTAGGGCAGATATATGAAGTCGTCCCCGAAAATAATTTGAGAGTTAGAGTAGGGGATGCGGCACAAGGAAAATTTAGTAAGGCGAGTTTCTTAAAGTACGTTAAGGACGGGACACAGGCGGAATTAACGGGAATCGCCGTAGTGCCCGAAAAATACGTATTCGCCACAGCAGCTTTGGCTACAGCGGCGCAGGAGCCACCTAAGCAGCCACCAGCGCAAGTGGTGGAACCACCGGAAACCGATATAGGGGTAGTGCCGGAATCTGAGACTCTCACACCAAATAAGTTGGTTTTCGAGAAAGATCCAGACAAGTTCTTGAAGACTATGGGCAAGGGAATAGCTTTGGACTTGACGGGAGTTACCCACAAACCGAAAGTTATTAACGAGCCAGGGAAAGTATCAGTAGAGGTGGCAATGAAGATAAATGCCGCATTGATGGAGCTGTGTAAGAAGGTTATGGGCGCCGATGACGCAGCAACTAAGACAAAATTCTTCTTGTACGTGATGCAGATTGCTTGCACGTTCTTTACATCGTCTTCGACGGAGTTCAAAGAGTTTGACTACATAGAAACCGATGATGGAAAGAAGATATATGCGGTGTGGGTATATGATTGCATTAAACAAGCTGCTGCTTCGACGGGTTATGAAAACCCGGTAAGGCAGTATCTAGCGTACTTCACACCAACCTTCATCACGGCGACCCTGAATGGTAAACTAGTGATGAACGAGAAGGTTATGGCACAGCATGGAGTACCACCGAAATTCTTTCCGTACACGATAGACTGCGTTCGTCCGACGTACGATCTGTTCAACAACGACGCAATACTAGCATGGAATTTAGCTAGACAGCAGGCGTTTAGAAACAAGACGGTAACGGCCGATAACACCTTACACAACGTCTTCCAACTATTGCAAAAGAAGTAG

>GLRaV-3 20Z_BA

ATGGCATTTGAACTGAAATTAGGGCAGATATATGAAGTCGTCCCCGAAAATAATTTGAGAGTTAGAGTAGGGGATGCGGCACAAGGAAAATTTAGTAAGGCGAGTTTCTTAAAGTACGTTAAGGACGGGACACAGGCGGAATTAACGGGAATCGCCGTAGTGCCCGAAAAATACGTATTCGCCACAGCAGCTTTGGCTACAGCGGCGCAGGAGCCACCTAGGCAGCCACCAGCGCAAGTGGTGGAACCACAGGAAACCGATATAGGGGTAGTGCCGGAATCTGAGACTCTCACACCAAATAAGTTGGTTTTCGAGAAAGATCCAGACAAGTTCTTGAAGACTATGGGCAAGGGAATAGCTTTGGACTTGACGGGAGTTACCCACAAACCGAAAGTTATTAACGAGCCAGGGAAAGTATCAGTAGATGTGGCAATGAAGATAAATGCCGCATTGATGGAGCTGTGTAAGAAGGTTATGGGCGCCGATGACGCAGCAACTAAGACAAAATTCTTCTTGTACGTGATGCAGATTGCTTGCACGTTCTTTACATCGTCTTCGACGGAGTTCAAAGAGTTTGACTACATAGAAACCGATGATGGAAAGAAGATATATGCGGTGTGGGTATATGATTGCATTAAACAAGCTGCTGCTTCGACGGGTTATGAAAACCCGGTAAGGCAGTATCTAGCGTACTTCACACCAACCTTCATCACGGCGACCCTGAATGGTAAACTAGTGATGAACGAGAAGGTTATGGCACAGCATGGAGTACCACCGAAATTCTTTCCGTACACGATAGACTGCGTTCGTCCGACGTACGATCTGTTCAACAACGACGCAATACTAGCATGGAATTTAGCTAGACAGCAGGCGTTTAGAAACAAGACGGTAACGGCCGATAACACCTTACATAACGTCTTCCAACTATTGCAAAAGAAGTAG

>GLRaV-3 21Z_BA

ATGGCATTTGAACTGAAATTAGGGCAGATATATGAAGTCGTCCCCGAGAATAATTTGAGAGTTAGAGTAGGGGATGCGGCACAAGGAAAATTTAGTAAGGCGAGTTTCTTAAAGTTCGTTAAGGACGGGACACAGGCGGAATTAACGGGAATCGCCGTAGTGCCCGAAAAATACGTATTCGCCACAGCAGCTTTAGCTACAGCGGCACAGGAGCCACCCAAGCAGCCCACCACGCAAGTGGTGGAACCCCCAGAAGCCGATATAGGGGTGGTGCCCGAATCCGAGACTCTTACCCCAAATAAGCTGGTTTTTGAAAAAGATCCAGACAAGTTCTTAAAGACTATGGGTAAGGGGATAGCTTTGGACTTAACGGGGGTTACCCATAAACCGAAAGTTATTAACGAGCCGGGAAAAGTATCAGTAGAGGTAGCAATGAAGATAAATGCCGCATTGATGGAGCTGTGTAAGAAGGTTATGGGCGCCGATGACGCAGCAACTAAGACAAAATTCTTCTTGTACGTGATGCAGATTGCTTGCACGTTCTTTACATCGTCTTCGACGGAGTTCAAAGAGTTTGACTACATAGAAACCGATGATGGAAAGAAGATATATGCGGTGTGGGTATATGATTGCATTAAACAAGCTGCTGCTTCGACGGGTTATGAAAACCCGGTAAGGCAGTATCTAGCGTACTTCACACCAACCTTCATCACGGCGACCCTGAATGGTAAACTAGTGATGAACGAGAAGGTTATGGCACAGCATGGAGTACCACCGAAATTCTTTCCGTACACGATAGACTGCGTTCGTCCGACGTACGATCTGTTCAACAACGACGCAATACTAGCATGGAATTTAGCTAGACAGCAGGCGTTTAGAAACAAGACGGTAACGGCCGATAACACCTTACACAACGTCTTCCAACTATTGCAAAAGAAGTAG

>GLRaV-3 24Z_BA

ATGGCATTTGAACTGAAATTAGGGCAGATATATGAAGTCGTCCCCGAAAATAATTTGAGAGTTAGAGTAGGGGATGCGGCACAAGGAAAATTTAGTAAGGCGAGTTTCTTAAAGTACGTTAAGGACGGGACACAGGCGGAATTAACGGGAATCGCCGTAGTGCCCGAAAAATACGTATTCGCCACAGCAGCTTTGGCTACAGCGGCGCAGGAGCCACCTAAGCAGCCACCAGCGCAAGTGGTGGAACCACAGGAAACCGATATAGGGGTGGTGCCGGAATCTGAGACTCTCACACCAAATAAGTTGGTTTTCGAGAAAGATCCAGACAAGTTCTTGAAGACTATGGGCAAGGGAATAGCTTTGGACTTGACGGGAGTTACCCACAAACCGAAAGTTATTAACGAGCCAGGGAAAGTATCAGTAGAGGTGGCAATGAAGATAAATGCCGCATTGATGGAGCTGTGTAAGAAGGTTATGGGCGCCGATGACGCAGCAACTAAGACAAAATTCTTCTTGTACGTGATGCAGATTGCTTGCACGTTCTTTACATCGTCTTCGACGGAGTTCAAAGAGTTTGACTACATAGAAACCGATGATGGAAAGAAGATATATGCGGTGTGGGTATATGATTGCATTAAACAAGCTGCTGCTTCGACGGGTTATGAAAACCCGGTAAGGCAGTATCTAGCGTACTTCACACCAACCTTCATCACGGCGACCCTGAATGGTAAACTAGTGATGAACGAGAAGGTTATGGCACAGCATGGAGTACCACCGAAATTCTTTCCGTACACGATAGACTGCGTTCGTCCGACGTACGATCTGTTCAACAACGACGCAATACTAGCATGGAATTTAGCTAGACAGCAGGCGTTTAGAAACAAGACGGTAACGGCCGATAACACCTTACACAACGTCTTCCAACTATTGCAAAAGAAGTAG

>GLRaV-3 25Z_BA

ATGGCATTTGAACTGAAATTAGGGCAGATATATGAAGTCGTCCCCGAAAATAATTTGAGAGTTAGAGTAGGGGATGCGGCACAAGGAAAATTTAGTAAGGCGAGTTTCTTAAAGTACGTTAAGGACGGGACACAGGCGGAATTAACGGGAATCGCCGTAGTGCCCGAAAAATACGTATTCGCCACAGCAGCTTTGGCTACAGCGGCGCAGGAGCCACCTAAGCAGCCGACAACGCAAGTGGTGGAACCACCAGAAGCCGATATAGGGGTGGTGCCGGAATCTGAGACTCTTACACCAAATAAGCTGGTTTTCGAGAAAGATCCAGACAAGTTCTTGAAGACTATGGGTAAGGGGATAGCTTTGGACTTGACGGGAGTTACCCATAAACCGAAAGTTATTAACGAGCCGGGAAAAGTATCAGTAGAGGTGGCAATGAAGATAAATGCCGCATTGATGGAGCTGTGTAAGAAGGTTATGGGCGCCGATGACGCAGCAACTAAGACAAAATTCTTCTTGTACGTGATGCAGATTGCTTGCACGTTCTTTACATCGTCTTCGACGGAGTTCAAAGAGTTTGACTACATAGAAACCGATGATGGAAAGAAGATATATGCGGTGTGGGTATATGATTGCATTAAACAAGCTGCTGCTTCGACGGGTTATGAAAACCCGGTAAGGCAGTATCTAGCGTACTTCACACCAACCTTCATCACGGCGACCCTGAATGGTAAACTAGTGATGAACGAGAAGGTTATGGCACAGCATGGAGTACCACCGAAATTCTTTCCGTACACGATAGACTGCGTTCGTCCGACGTACGATCTGTTCAACAACGACGCAATACTAGCATGGAATTTAGCTAGACAGCAGGCGTTTAGAAACAAGACGGTAACGGCCGATAACACCTTACACAACGTCTTCCAACTATTGCAAAAGAAGTAG

>GLRaV-3 26Z_BA

ATGGCATTTGAACTGAAATTAGGGCAGATATATGAAGTCGTCCCCGAGAATAATTTGAGAGTTAGAGTAGGGGATGCGGCACAAGGAAAATTTAGTAAGGCGAGTTTCTTAAAGTACGTTAAGGACGGGACACAGGCGGAATTAACGGGAATCGCCGTAGTGCCCGAAAAATACGTATTCGCCACAGCAGCTTTGGCTACAGCGGCGCAGGAGCCACCTAGGCAGCCACCAGCGCAAGTGGTGGAACCACAGGAAACCGATATAGGGGTAGTGCCGGAATCTGAGACTCTCACACCAAATAAGTTGGTTTTCGAGAAAGATCCAGACAAGTTCTTGAAGACTATGGGCAAGGGAATAGCTTTGGACTTGACGGGAGTTACCCACAAACCGAAAGTTATTAACGAGCCAGGGAAAGTATCAGTAGAGGTGGCAATGAAGATAAATGCCGCATTGATGGAGCTGTGTAAGAAGGTTATGGGCGCCGATGACGCAGCAACTAAGACAAAATTCTTCTTGTACGTGATGCAGATTGCTTGCACGTTCTTTACATCGTCTTCGACGGAGTTCAAAGAGTTTGACTACATAGAAACCGATGATGGAAAGAAGATATATGCGGTGTGGGTATATGATTGCATTAAACAAGCTGCTGCTTCGACGGGTTATGAAAACCCGGTAAGGCAGTATCTAGCGTACTTCACACCAACCTTCATCACGGCGACCCTGAATGGTAAACTAGTGATGAACGAGAAGGTTATGGCACAGCATGGAGTACCACCGAAATTCTTTCCGTACACGATAGACTGCGTTCGTCCGACGTACGATCTGTTCAACAACGACGCAATACTAGCATGGAATTTAGCTAGACAGCAGGCGTTTAGAAACAAGACGGTAACGGCCGATAACACCTTACACAACGTCTTCCAACTATTGCAAAAGAAGTAG

>GLRaV-3 27Z_BA

ATGGCATTTGAACTGAAATTAGGGCAGATATATGAAGTCGTCCCCGAGAATAATTTGAGAGTTAGAGTAGGGGATGCGGCACAAGGAAAATTTAGTAAGGCGAGTTTCTTAAAGTACGTTAAGGACGGGACACAGGCGGAATTAACGGGAATCGCCGTAGTGCCCGAAAAATACGTATTCGCCACAGCAGCTTTAGCTACAGCGGCACAGGAGCCACCTAAGCAGCCAACAACGCAAGTGGTGGAACCACAGGAAACCGATATAGGGGTGGTGCCGGAATCTGAGACTCTCACACCAAATAAGTTGGTTTTCGAGAAAGATCCAGACAAGTTCTTAAAGACTATGGGCAAGGGAATAGCTTTGGACTTAACGGGAGTTACCCACAAACCGAAAGTTATTAACGAGCCAGGGAAAGTATCAGTAGAGGTGGCAATGAAGATAAATGCCGCATTGATGGAGCTGTGTAAGAAGGTTATGGGCGCCGATGACGCAGCAACTAAGACAAAATTCTTCTTGTACGTGATGCAGATTGCTTGCACGTTCTTTACATCGTCTTCGACGGAGTTCAAAGAGTTTGACTACATAGAAACCGATGATGGAAAGAAGATATATGCGGTGTGGGTATATGATTGCATTAAACAAGCTGCTGCTTCGACGGGTTATGAAAACCCGGTAAGGCAGTATCTAGCGTACTTCACACCAACCTTCATCACGGCGACCCTGAATGGTAAACTAGTGATGAACGAGAAGGTTATGGCACAGCATGGAGTACCACCGAAATTCTTTCCGTACACGATAGACTGCGTTCGTCCGACGTACGATCTGTTCAACAACGACGCAATACTAGCATGGAATTTAGCTAGACAGCAGGCGTTTAGAAACAAGACGGTAACGGCCGATAACACCTTACACAACGTCTTCCAACTATTGCAAAAGAAGTAG

>GLRaV-3 28Z_BA

ATGGCATTTGAACTGAAATTAGGGCAGATATATGAAGTCGTCCCCGAAAATAATTTGAGAGTTAGAGTAGGGGATGCGGCACAAGGAAAATTTAGTAAGGCGAGTTTCTTAAAGTACGTTAAGGACGGGACACAGGCGGAATTAACGGGAATCGCCGTAGTGCCCGAAAAATACGTATTCGCCACAGCAGCTTTAGCTACAGCGGCACAGGAGCCACCTAGGCAGCCACCAACGCAAGTGGTGGAACCACAGGAAACCGATATAGGGGTAGTGCCGGAATCTGAGACTCTCACACCAAATAAGTTGGTTTTCGAGAAAGATCCAGACAAGTTCTTAAAGACTATGGGCAAGGGAATAGCTTTGGACTTAACGGGAGTTACCCACAAACCGAAAGTTATTAACGAGCCAGGGAAAGTATCAGTAGAGGTGGCAATGAAGATAAATGCCGCATTGATGGAGCTGTGTAAGAAGGTTATGGGCGCCGATGACGCAGCAACTAAGACAAAATTCTTCTTGTACGTGATGCAGATTGCTTGCACGTTCTTTACATCGTCTTCGACGGAGTTCAAAGAGTTTGACTACATAGAAACCGATGATGGAAAGAAGATATATGCGGTGTGGGTATATGATTGCATTAAACAAGCTGCTGCTTCGACGGGTTATGAAAACCCGGTAAGGCAGTATCTAGCGTACTTCACACCAACCTTCATCACGGCGACCCTGAATGGTAAACTAGTGATGAACGAGAAGGTTATGGCACAGCATGGAGTACCACCGAAATTCTTTCCGTACACGATAGACTGCGTTCGTCCGACGTACGATCTGTTCAACAACGACGCAATACTAGCATGGAATTTAGCTAGACAGCAGGCGTTTAGAAACAAGACGGTAACGGCCGATAACACCTTACACAACGTCTTCCAACTATTGCAAAAGAAGTAG

>GLRaV-3 29Z_BA

ATGGCATTTGAACTGAAATTAGGGCAGATATATGAAGTCGTCCCCGAAAATAATTTGAGAGTTAGAGTAGGGGATGCGGCACAAGGAAAATTTAGTAAGGCGAGTTTCTTAAAGTACGTTAAGGACGGGACACAGGCGGAATTAACGGGAATCGCCGTAGTGCCCGAAAAATACGTATTCGCTACAGCAGCTTTGGCTACAGCGGCGCAGGAGCCACCTACGCAGCCACCAGCGCAAGTGGTGGAACCACAGGAAACCGATATAGGGGTAGTGCCGGAATCTGAAACTCTCACACCAAATAAGTTGGTTTTTGAGAAAGATCCAGACAAGTTCTTGAAGACTATGGGCAAGGGAATAGCTTTGGACTTGACGGGAGTTACCCACAAACCGAAAGTTATTAACGAGCCAGGGAAAGTATCAGTAGAGGTGGCAATGAAGATAAATGCCGCATTGATGGAGCTGTGTAAGAAGGTTATGGGCGCCGATGACGCAGCAACTAAGACAAAATTCTTCTTGTACGTGATGCAGATTGCTTGCACGTTCTTTACATCGTCTTCGACGGAGTTCAAAGAGTTTGACTACATAGAAACCGATGATGGAAAGAAGATATATGCGGTGTGGGTATATGATTGCATTAAACAAGCTGCTGCTTCGACGGGTTATGAAAACCCGGTAAGGCAGTATCTAGCGTACTTCACACCAACCTTCATCACGGCGACCCTGAATGGTAAACTAGTGATGAACGAGAAGGTTATGGCACAGCATGGAGTACCACCGAAATTCTTTCCGTACACGATAGACTGCGTTCGTCCGACGTACGATCTGTTCAACAACGACGCAATACTAGCATGGAATTTAGCTAGACAGCAGGCGTTTAGAAACAAGACGGTAACGGCCGATAACACCTTACACAACGTCTTCCAACTATTGCAAAAGAAGTAG

>GLRaV-3 30Z_BA

ATGGCATTTGAACTGAAATTAGGGCAGATATATGAAGTCGTCCCCGAAAATAATTTGAGAGTTAGAGTAGGGGATGCGGCACAAGGAAAATTTAGTAAGGCGAGTTTCTTAAAGTACGTTAAGGACGGGACACAGGCGGAATTAACGGGAATCGCCGTAGTGCCCGAAAAATACGTATTCGCCACAGCAGCTTTGGCTACAGCGGCGCAGGAGCCACCTAGGCAGCCACCAGCGCAAGTGGTGGAACCACAGGAAACCGATATAGGGGTAGTGCCGGAATCTGAGACTCTCACACCAAATAAGTTGGTTTTCGAGAAAGATCCAGACAAGTTCTTGAAGACTATGGGCAAGGGAATAGCTTTGGACTTGACGGGAGTTACCCACAAACCGAAAGTTATTAACGAGCCAGGGAAAGTATCAGTAGAGGTGGCAATGAAGATAAATGCCGCATTGATGGAGCTGTGTAAGAAGGTTATGGGCGCCGATGACGCAGCAACTAAGACAAAATTCTTCTTGTACGTGATGCAGATTGCTTGCACGTTCTTTACATCGTCTTCGACGGAGTTCAAAGAGTTTGACTACATAGAAACCGATGATGGAAAGAAGATATATGCGGTGTGGGTATATGAATGCATTAAACAAGCTGCTGCTTCGACGGGTTATGAAAACCCGGTAAGGCAGTATCTAGCGTACTTCACACCAACCTTCATCACGGCGACCCTGAATGGTAAATTAGTGATGAACGAGAAGGTTATGGCACAGCATGGAGTACCACCGAAATTCTTTCCGTACACGATAGACTGCGTTGGTCCAACGTACGATCTGTTCAACAACGACGCAATACTAGCATGGAATTTAGCTAGACAGCAGGCGTTTAGAAACAAGACGGTAACGGCCGATAACACCTTACACAACGTCTTCCAACTATTGCAAAAGAAGTAG

>GLRaV-3 43B_BA

ATGGCATTTGAACTGAAATTAGGGCAGATATATGAAGTCGTCCCCGAAAATAATTTGAGAGTTAGAGTAGGGGATGCGGCACAAGGAAAATTTAGTAAGGCGAGTTTCTTAAAGTACGTTAAGGACGGGACACAGGCGGAATTAACGGGAATCGCCGTAGTGCCCGAAAAATACGTATTCGCCACAGCAGCTTTGGCTACAGCGGCGCAGGAGCCACCTAGGCAGCCACCAGCGCAAGTGGTGGAACCACAGGAAACCGATATAGGGGTAGTGCCGGAATCTGAGACTCTCACACCAAATAAGTTGGTTTTCGAGAAAGATCCAGACAAGTTCTTGAAGACTATGGGCAAGGGAATAGCTTTGGACTTGACGGGAGTTACCCACAAACCGAAAGTTATTAACGAGCCAGGGAAAGTATCAGTAGAGGTGGCAATGAAGATAAATGCCGCATTGATGGAGCTGTGTAAGAAGGTTATGGGCGCCGATGACGCAGCAACTAAGACAAAATTCTTCTTGTACGTGATGCAGATTGCTTGCACGTTCTTTACATCGTCTTCGACGGAGTTCAAAGAGTTTGACTACATAGAAACCGATGATGGAAAGAAGATATATGCGGTGTGGGTATATGATTGCATTAAACAAGCTGCTGCTTCGACGGGTTATGAAAACCCGGTAAGGCAGTATCTAGCGTACTTCACACCAACCTTCATCACGGCGACCCTGAATGGTAAACTAGTGATGAACGAGAAGGTTATGGCACAGCATGGAGTACCACCGAAATTCTTTCCGTACACGATAGACTGCGTTCGTCCGACGTACGATCTGTTCAACAACGACGCAATACTAGCATGGAATTTAGCTAGACAGCAGGCGTTTAGAAACAAGACGGTAACGGCCGATAACACCTTACACAACGTCTTCCAACTATTGCAAAAGAAGTAG

>GLRaV-3 45B_BA

ATGGCATTTGAACTGAAATTAGGGCAGATATATGAAGTCGTCCCCGAAAATAATTTGAGAGTTAGAGTAGGGGATGCGGCACAAGGAAAATTTAGTAAGGCGAGTTTCTTAAAGTACGTTAAGGACGGGACACAGGCGGAATTAACGGGAATCGCCGTAGTGCCCGAAAAATACGTATTCGCCACAGCGGCTTTGGCTACAGCGGCGCAAGAGCCACCTACGCAGCCGCCAGCGCAGGTGGTGGAACCACAGGAAACCGATATAGGGGTGGTGCCGGAATCTGAAACTCTCACACCAAATAAGTTGGTTTTCGAGAAAGATCCAAACAAGTTCTTGAAGACTATGGGCAAGGGAATAGCTTTGGACTTGACGGGAGTTACCCACAAACCGAAAGTTATTAACGAGCCGGGGAAAGTATCAGTAGAGGTGGCAATGAAGATAAATGCCGCATTGATGGAGCTGTGTAAGAAGGTTATGGGCGCCGATGACGCAGCAACTAAGACAAAATTCTTCTTGTACGTGATGCAGATTGCTTGCACGTTCTTTACATCGTCTTCGACGGAGTTCAAAGAGTTTGACTACATAGAAACCGATGATGGAAAGAAGATATATGCGGTGTGGGTATATGATTGCATTAAACAAGCTGCTGCTTCGACGGGTTATGAAAACCCGGTAAGGCAGTATCTAGCGTACTTCACACCAACCTTCATCACGGCGACCCTGAATGGTAAACTAGTGATGAACGAGAAGGTTATGGCACAGCATGGAGTACCACCGAAATTCTTTCCGTACACGATAGACTGCGTTCGTCCGACGTACGATCTGTTCAACAACGACGCAATACTAGCATGGAATTTAGCTAGACAGCAGGCGTTTAGAAACAAGACGGTAACGGCCGATAACACCTTACACAACGTCTTCCAACTATTGCAAAAGAAGTAG

>GLRaV-3 48B_BA

ATGGCATTTGAACTGAAATTAGGGCAGATATATGAAGTCGTCCCCGAGAATAATTTGAGAGTTAGAGTAGGGGATGCGGCACAAGGAAAATTTAGTAAGGCGAGTTTCTTAAAGTACGTTAAGGACGGGACACAGGCGGAATTAACGGGAATCGCCGTAGTGCCCGAAAAATACGTATTCGCCACAGCAGCTTTAGCTACAGCGGCGCAGGAGCCACCTAAGCAGCCGACAACGCAAGTGGTGGAACCACCAGAAACCGATATAGGGGTGGTGCCGGAATCTGAGACTCTCACACCAAATAAGCTGGTTTTTGAGAAAGATCCAGACAAGTTCTTGAAGACTATGGGTAAGGGGATAGCTTTGGACTTGACGGGAGTTACCCACAAACCGAAAGTTATTAACGAGCCGGGAAAAGTATCAGTAGAGGTGGCAATGAAGATAAATGCCGCATTGATGGAGCTGTGTAAGAAGGTTATGGGCGCCGATGACGCAGCAACTAAGACAAAATTCTTCTTGTACGTGATGCAGATTGCTTGCACGTTCTTTACATCGTCTTCGACGGAGTTCAAAGAGTTTGACTACATAGAAACCGATGATGGAAAGAAGATATATGCGGTGTGGGTATATGATTGCATTAAACAAGCTGCTGCTTCGACGGGTTATGAAAACCCGGTAAGGCAGTATCTAGCGTACTTCACACCAACCTTCATCACGGCGACCCTGAATGGTAAACTAGTGATGAACGAGAAGGTTATGGCACAGCATGGAGTACCACCGAAATTCTTTCCGTACACGATAGACTGCGTTCGTCCGACGTACGATCTGTTCAACAACGACGCAATACTAGCATGGAATTTAGCTAGACAGCAGGCGTTTAGAAACAAGACGGTAACGGCCGATAACACCTTACATAACGTCTTCCAACTATTGCAAAAGAAGTAG

>GLRaV-3 52B_BA

ATGGCATTTGAACTGAAATTAGGGCAGATATATGAAGTCGTCCCCGAGAATAATTTGAGAGTTAGAGTAGGGGATGCGGCACAAGGAAAATTTAGTAAGGCGAGTTTCTTAAAGTACGTTAAGGACGGGACACAGGCGGAATTAACGGGAATCGCCGTAGTGCCCGAAAAATACGTATTCGCCACAGCAGCTTTGGCTACAGCGGCGCAGGAGCCACCTAGGCAGCCACCAGCGCAAGTGGTGGAACCACAGGAAACCGATATAGGGGTAGTGCCGGAATCTGAGACTCTCACACCAAATAAGTTGGTTTTCGAGAAAGATCCAGACAAGTTCTTGAAGACTATGGGCAAGGGAATAGCTTTGGACTTGACGGGAGTTACCCACAAACCGAAAGTTATTAACGAGCCAGGGAAAGTATCAGTAGAGGTGGCAATGAAGATAAATGCCGCATTGATGGAGCTGTGTAAGAAGGTTATGGGCGCCGATGACGCAGCAACTAAGACAAAATTCTTCTTGTACGTGATGCAGATTGCTTGCACGTTCTTTACATCGTCTTCGACGGAGTTCAAAGAGTTTGACTACATAGAAACCGATGATGGAAAGAAGATATATGCGGTGTGGGTATATGATTGCATTAAACAAGCTGCTGCTTCGACGGGTTATGAAAACCCGGTAAGGCAGTATCTAGCGTACTTCACACCAACCTTCATCACGGCGACCCTGAATGGTAAACTAGTGATGAACGAGAAGGTTATGGCACAGCATGGAGTACCACCGAAATTCTTTCCGTACACGATAGACTGCGTTCGTCCGACGTACGATCTGTTCAACAACGACGCAATACTAGCATGGAATTTAGCTAGACAGCAGGCGTTTAGAAACAAGACGGTAACGGCCGATAACACCTTACACAACGTCTTCCAACTATTGCAAAAGAAGTAG

>GLRaV-3 55B_BA

ATGGCATTTGAACTGAAATTAGGGCAGATATATGAAGTCGTCCCCGAAAATAATTTGAGAGTTAGAGTAGGGGATGCGGCACAAGGAAAATTTAGTAAGGCGAGTTTCTTAAAGTACGTTAAGGACGGGACACAGGCGGAATTAACGGGAATCGCCGTAGTGCCCGAAAAATACGTATTCGCCACAGCAGCTTTAGCTACAGCGGCACAGGAGCCACCTAAGCAGCCGACAACGCAAGTGGTGGAACCACCAGAAGCCGATATAGGGGTGGTGCCGGAATCTGAGACTCTTACACCAAATAAGCTGGTTTTTGAGAAAGATCCAGACAAGTTCTTGAAGACTATGGGTAAGGGGATAGCTTTGGACTTGACGGGGGTTACCCATAAACCGAAAGTTATTAACGAGCCGGGAAAAGTATCAGTAGAGGTGGCAATGAAGATAAATGCCGCATTGATGGAGCTGTGTAAGAAGGTTATGGGCGCCGATGACGCAGCAACTAAGACAAAATTCTTCTTGTACGTGATGCAGATTGCTTGCACGTTCTTTACATCGTCTTCGACGGAGTTCAAAGAGTTTGACTACATAGAAACCGATGATGGAAAGAAGATATATGCGGTGTGGGTATATGATTGCATTAAACAAGCTGCTGCTTCGACGGGTTATGAAAACCCGGTAAGGCAGTATCTAGCGTACTTCACACCAACCTTCATCACGGCGACCCTGAATGGTAAACTAGTGATGAACGAGAAGGTTATGGCACAGCATGGAGTACCACCGAAATTCTTTCCGTACACGATAGACTGCGTTCGTCCGACGTACGATCTGTTCAACAACGACGCAATACTAGCATGGAATTTAGCTAGACAGCAGGCGTTTAGAAACAAGACGGTAACGGCCGATAACACCTTACACAACGTCTTCCAACTATTGCAAAAGAAGTAG

>GLRaV-3 56B_BA

ATGGCATTTGAACTGAAATTAGGGCAGATATATGAAGTCGTCCCCGAAAATAATTTGAGAGTTAGAGTAGGGGATGCGGCACAAGGAAAATTTAGTAAGGCGAGTTTCTTAAAGTACGTTAAGGACGGGACACAGGCGGAATTAACGGGAATCGCCGTAGTGCCCGAAAAATACGTATTCGCCACAGCAGCTTTGGCTACAGCGGCGCAGGAGCCACCTAAGCAGCCGCCAGCGCAAGTGGTGGAACCACAGGAAACCGATATAGGGGTGGTGCCGGAATCTGAGACTCTCACACCAAATAAGTTGGTTTTCGAGAAAGATCCAGACAAGTTCTTGAAGACTATGGGCAAGGGAATAGCTTTGGACTTGACGGGAGTTACCCACAAACCGAAAGTTATTAACGAGCCAGGGAAAGTATCAGTAGAGGTGGCAATGAAGATAAATGCCGCATTGATGGAGCTGTGTAAGAAGGTTATGGGCGCCGATGACGCAGCAACTAAGACAAAATTCTTCTTGTACGTGATGCAGATTGCTTGCACGTTCTTTACATCGTCTTCGACGGAGTTCAAAGAGTTTGACTACATAGAAACCGATGATGGAAAGAAGATATATGCGGTGTGGGTATATGATTGCATTAAACAAGCTGCTGCTTCGACGGGTTATGAAAACCCGGTAAGGCAGTATCTAGCGTACTTCACACCAACCTTCATCACGGCGACCCTGAATGGTAAACTAGTGATGAACGAGAAGGTTATGGCACAGCATGGAGTACCACCGAAATTCTTTCCGTACACGATAGACTGCGTTCGTCCGACGTACGATCTGTTCAACAACGACGCAATACTAGCATGGAATTTAGCTAGACAGCAGGCGTTTAGAAACAAGACGGTAACGGCCGATAACACCTTACACAACGTCTTCCAACTATTGCAAAAGAAGTAG

>GLRaV-3 57B_BA

ATGGCATTTGAACTGAAATTAGGGCAGATATATGAAGTCGTCCCCGAAAATAATTTGAGAGTTAGAGTAGGGGATGCGGCACAAGGAAAATTTAGTAAGGCGAGTTTCTTAAAGTACGTTAAGGACGGGACACAGGCGGAATTAACGGGAATCGCCGTAGTGCCCGAAAAATACGTATTCGCCACAGCAGCTTTGGCTACAGCGGCACAGGAGCCACCTAAGCAGCCGACAACGCAAGTGGTGGAACCACCAGAAGCCGATATAGGGGTGGTGCCGGAATCTGAGACTCTTACACCAAATAAGCTGGTTTTTGAGAAAGATCCAGACAAGTTCTTGAAGACTATGGGTAAGGGGATAGCTTTGGACTTGACGGGGGTTACCCATAAACCGAAAGTTATTAACGAGCCGGGAAAAGTATCAGTAGAGGTAGCAATGAAGATAAATGCCGCATTGATGGAGCTGTGTAAGAAGGTTATGGGCGCCGATGACGCAGCAACTAAGACAAAATTCTTCTTGTACGTGATGCAGATTGCTTGCACGTTCTTTACATCGTCTTCGACGGAGTTCAAAGAGTTTGACTACATAGAAACCGATGATGGAAAGAAGATATATGCGGTGTGGGTATATGATTGCATTAAACAAGCTGCTGCTTCGACGGGTTATGAAAACCCGGTAAGGCAGTATCTAGCGTACTTCACACCAACCTTCATCACGGCGACCCTGAATGGTAAACTAGTGATGAACGAGAAGGTTATGGCACAGCATGGAGTACCACCGAAATTCTTTCCGTACACGATAGACTGCGTTCGTCCGACGTACGATCTGTTCAACAACGACGCAATACTAGCATGGAATTTAGCTAGACAGCAGGCGTTTAGAAACAAGACGGTAACGGCCGATAACACCTTACACAACGTCTTCCAACTATTGCAAAAGAAGTAG

>GLRaV-3 60B_BA

ATGGCATTTGAACTGAAATTAGGGCAGATATATGAAGTCGTCCCCGAAAATAATTTGAGAGTTAGAGTAGGGGATGCGGCACAAGGAAAATTTAGTAAGGCGAGTTTCTTAAAGTACGTTAAGGACGGGACACAGGCGGAATTAACGGGAATCGCCGTAGTGCCCGAAAAATACGTATTCGCCACAGCAGCTTTGGCTACAGCGGCGCAGGAGCCACCTAGGCAGCCACCAGCGCAAGTGGTGGAACCACAGGAAACCGATATAGGGGTGGTGCCGGAATCTGAGACTCTCACACCAAATAAGTTGGTTTTCGAGAAAGATCCAGACAAGTTCTTGAAGACTATGGGCAAGGGAATAGCTTTGGACTTGACGGGAGTTACCCACAAACCGAAAGTTATTAACGAGCCAGGGAAAGTATCAGTAGAGGTGGCAATGAAGATAAATGCCGCATTGATGGAGCTGTGTAAGAAGGTTATGGGCGCCGATGACGCAGCAACTAAGACAAAATTCTTCTTGTACGTGATGCAGATTGCTTGCACGTTCTTTACATCGTCTTCGACGGAGTTCAAAGAGTTTGACTACATAGAAACCGATGATGGAAAGAAGATATATGCGGTGTGGGTATATGATTGCATTAAACAAGCTGCTGCTTCGACGGGTTATGAAAACCCGGTAAGGCAGTATCTAGCGTACTTCACACCAACCTTCATCACGGCGACCCTGAATGGTAAACTAGTGATGAACGAGAAGGTTATGGCACAGCATGGAGTACCACCGAAATTCTTTCCGTACACGATAGACTGCGTTCGTCCGACGTACGATCTGTTCAACAACGACGCAATACTAGCATGGAATTTAGCTAGACAGCAGGCGTTTAGAAACAAGACGGTAACGGCCGATAACACCTTACACAACGTCTTCCAACTATTGCAAAAGAAGTAG

>GLRaV-3 65B_BA

ATGGCATTTGAACTGAAATTAGGGCAGATATATGAAGTCGTCCCCGAAAATAATTTGAGAGTTAGAGTAGGGGATGCGGCACAAGGAAAATTTAGTAAGGCGAGTTTCTTAAAGTACGTTAAGGACGGGACACAGGCGGAATTAACGGGAATCGCCGTAGTGCCCGAAAAATACGTATTCGCCACAGCAGCTTTAGCTACAGCGGCACAGGAGCCACCTAAGCAGCCGACAACGCAAGTGGTGGAACCACCAGAAGCCGATATAGGGGTGGTGCCGGAATCTGAGACTCTTACACCAAATAAGCTGGTTTTTGAGAAAGATCCAAACAAGTTCTTGAAGACTATGGGCAAGGGAATAGCTTTGGACTTGACTGGAGTTACCCACAAACCGAAAGTTATTAACGAGCCAGGGAAAGTATCAGTAGAGGTGGCAATGAAGATAAATGCCGCATTGATGGAGCTGTGTAAGAAGGTTATGGGCGCCGATGACGCAGCAACTAAGACAAAATTCTTCTTGTACGTGATGCAGATTGCTTGCACGTTCTTTACATCGTCTTCGACGGAGTTCAAAGAGTTTGACTACATAGAAACCGATGATGGAAAGAAGATATATGCGGTGTGGGTATATGATTGCATTAAACAAGCTGCTGCTTCGACGGGTTATGAAAACCCGGTAAGGCAGTATCTAGCGTACTTCACACCAACCTTCATCACGGCGACCCTGAATGGTAAACTAGTGATGAACGAGAAGGTTATGGCACAGCATGGAGTACCACCGAAATTCTTTCCGTACACGATAGACTGCGTTCGTCCGACGTACGATCTGTTCAACAACGACGCAATACTAGCATGGAATTTAGCTAGACAGCAGGCGTTTAGAAACAAGACGGTAACGGCCGATAACACCTTACACAACGTCTTCCAACTATTGCAAAAGAAGTAG

>GLRaV-3 66B_BA

ATGGCATTTGAACTGAAATTAGGGCAGATATATGAAGTCGTCCCCGAGAATAATTTGAGAGTTAGAGTAGGGGATGCGGCACAAGGAAAATTTAGTAAGGCGAGTTTCTTAAAGTACGTTAAGGACGGGACACAGGCGGAATTAACGGGAATCGCCGTAGTGCCCGAAAAATACGTATTCGCCACAGCAGCTTTGGCTACAGCGGCGCAGGAGCCACCTAGGCAGCCACCGGCGCAAGTGGTGGAACCACAGGAAACCGATATAGGGGTAGTGCCGGAATCTGAGACTCTCACACCAAATAAGTTGGTTTTCGAGAAAGATCCAGACAAGTTCTTGAAGACTATGGGCAAGGGAATAGCTTTGGACTTGACGGGAGTTACCCACAAACCGAAAGTTATTAACGAGCCAGGGAAAGTATCAGTAGAGGTGGCAATGAAGATAAATGCCGCATTGATGGAGCTGTGTAAGAAGGTTATGGGCGCCGATGACGCAGCAACTAAGACAAAATTCTTCTTGTACGTGATGCAGATTGCTTGCACGTTCTTTACATCGTCTTCGACGGAGTTCAAAGAGTTTGACTACATAGAAACCGATGATGGAAAGAAGATATATGCGGTGTGGGTATATGATTGCATTAAACAAGCTGCTGCTTCGACGGGTTATGAAAACCCGGTAAGGCAGTATCTAGCGTACTTCACACCAACCTTCATCACGGCGACCCTGAATGGTAAACTAGTGATGAACGAGAAGGTTATGGCACAGCATGGAGTACCACCGAAATTCTTTCCGTACACGATAGACTGCGTTCGTCCGACGTACGATCTGTTCAACAACGACGCAATACTAGCATGGAATTTAGCTAGACAGCAGGCGTTTAGAAACAAGACGGTAACGGCCGATAACACCTTACACAACGTCTTCCAACTATTGCAAAAGAAGTAG

>GLRaV-3 74B_BA

ATGGCATTTGAACTGAAATTAGGGCAGATATATGAAGTCGTCCCCGAAAATAATTTGAGAGTTAGAGTAGGGGATGCGGCACAAGGAAAATTTAGTAAGGCGAGTTTCTTAAAGTACGTTAAGGACGGGACACAGGCGGAATTAACGGGAATCGCCGTAGTGCCCGAAAAATACGTATTCGCCACAGCAGCTTTGGCTACAGCGGCGCAGGAGCCACCTAAGCAGCCACCAGCGCAAGTGGTGGAACCACAGGAAACCGATATAGGGGTGGTGCCGGAATCTGAGACTCTCACACCAAATAAGTTGGTTTTCGAGAAAGATCCAGACAAGTTCTTGAAGACTATGGGCAAGGGAATAGCTTTGGACTTGACGGGAGTTACCCACAAACCGAAAGTTATTAACGAGCCAGGGAAAGTATCAGTAGAGGTGGCAATGAAGATAAATGCCGCATTGATGGAGCTGTGTAAGAAGGTTATGGGCGCCGATGACGCAGCAACTAAGACAAAATTCTTCTTGTACGTGATGCAGATTGCTTGCACGTTCTTTACATCGTCTTCGACGGAGTTCAAAGAGTTTGACTACATAGAAACCGATGATGGAAAGAAGATATATGCGGTGTGGGTATATGATTGCATTAAACAAGCTGCTGCTTCGACGGGTTATGAAAACCCGGTAAGGCAGTATCTAGCGTACTTCACACCAACCTTCATCACGGCGACCCTGAATGGTAAACTAGTGATGAACGAGAAGGTTATGGCACAGCATGGAGTACCACCGAAATTCTTTCCGTACACGATAGACTGCGTTCGTCCGACGTACGATCTGTTCAACAACGACGCAATACTAGCATGGAATTTAGCTAGACAGCAGGCGTTTAGAAACAAGACGGTAACGGCCGATAACACCTTACACAACGTCTTCCAACTATTGCAAAAGAAGTAG

>GLRaV-3 75B_BA

ATGGCATTTGAACTGAAATTAGGGCAGATATATGAAGTCGTCCCCGAAAATAATTTGAGAGTTAGAGTAGGGGATGCGGCACAAGGAAAATTTAGTAAGGCGAGTTTCTTAAAGTACGTTAAGGACGGGACACAGGCGGAATTAACGGGAATCGCCGTAGTGCCCGAAAAATACGTATTCGCCACAGCAGCTTTGGCTACAGCGGCGCAGGAGCCACCTAAGCAGCCGCCAGCGCAAGTGGTGGAACCACAGGAAACCGATATAGGGGTGGTGCCGGAATCTGAGACTCTCACACCAAATAAGTTGGTTTTCGAGAAAGATCCAGACAAGTTCTTGAAGACTATGGGCAAGGGAATAGCTTTGGACTTGACGGGAGTTACCCACAAACCGAAAGTTATTAACGAGCCAGGGAAAGTATCAGTAGAGGTGGCAATGAAGATAAATGCCGCATTGATGGAGCTGTGTAAGAAGGTTATGGGCGCCGATGACGCAGCAACTAAGACAAAATTCTTCTTGTACGTGATGCAGATTGCTTGCACGTTCTTTACATCGTCTTCGACGGAGTTCAAAGAGTTTGACTACATAGAAACCGATGATGGAAAGAAGATATATGCGGTGTGGGTATATGATTGCATTAAACAAGCTGCTGCTTCGACGGGTTATGAAAACCCGGTAAGGCAGTATCTAGCGTACTTCACACCAACCTTCATCACGGCGACCCTGAATGGTAAACTAGTGATGAACGAGAAGGTTATGGCACAGCATGGAGTACCACCGAAATTCTTTCCGTACACGATAGACTGCGTTCGTCCGACGTACGATCTGTTCAACAACGACGCAATACTAGCATGGAATTTAGCTAGACAGCAGGCGTTTAGAAACAAGACGGTAACGGCCGATAACACCTTACACAACGTCTTCCAACTATTGCAAAAGAAGTAG

>GLRaV-3 77B_BA

ATGGCATTTGAACTGAAATTAGGGCAGATATATGAAGTCGTCCCCGAAAATAATTTGAGAGTTAGAGTAGGGGATGCGGCACAAGGAAAATTTAGTAAGGCGAGTTTCTTAAAGTACGTTAAGGACGGGACACAGGCGGAATTAACGGGAATCGCCGTAGTGCCCGAAAAATACGTATTCGCCACAGCAGCTTTGGCTACAGCGGCGCAGGAGCCACCTAGGCAGCCACCAGCGCAAGTGGTGGAACCACAGGAAACCGATATAGGGGTAGTGCCGGAATCTGAGACTCTCACACCAAATAAGTTGGTTTTCGAGAAAGATCCAGACAAGTTCTTGAAGACTATGGGCAAGGGAATAGCTTTGGACTTGACGGGAGTTACCCACAAACCGAAAGTTATTAACGAGCCAGGGAAAGTATCAGTAGAGGTGGCAATGAAGATAAATGCCGCATTGATGGAGCTGTGTAAGAAGGTTATGGGCGCCGATGACGCAGCAACTAAGACAAAATTCTTCTTGTACGTGATGCAGATTGCTTGCACGTTCTTTACATCGTCTTCGACGGAGTTCAAAGAGTTTGACTACATAGAAACCGATGATGGAAAGAAGATATATGCGGTGTGGGTATATGATTGCATTAAACAAGCTGCTGCTTCGACGGGTTATGAAAACCCGGTAAGGCAGTATCTAGCGTACTTCACACCAACCTTCATCACGGCGACCCTGAATGGTAAACTAGTGATGAACGAGAAGGTTATGGCACAGCATGGAGTACCACCGAAATTCTTTCCGTACACGATAGACTGCGTTCGTCCGACGTACGATCTGTTCAACAACGACGCAATACTAGCATGGAATTTAGCTAGACAGCAGGCGTTTAGAAACAAGACGGTAACGGCCGATAACACCTTACACAACGTCTTCCAACTATTGCAAAAGAAGTAG

>GLRaV-3 78B_BA

ATGGCATTTGAACTGAAATTAGGGCAGATATATGAAGTCGTCCCCGAAAATAATTTGAGAGTTAGAGTAGGGGATGCGGCACAAGGAAAATTTAGTAAGGCGAGTTTCTTAAAGTACGTTAAGGACGGGACACAGGCGGAATTAACGGGAATCGCCGTAGTGCCCGAAAAATACGTATTCGCCACAGCAGCTTTGGCTACAGCGGCGCAGGAGCCACCTAAGCAGCCACCAACGCAAGTGGTGGAACCACAAGAAACCGATATAGGGGTGGTGCCGGAATCTGAGACTCTCACACCAAATAAGTTGGTTTTCGAGAAAGATCCAGACAAGTTCTTGAAGACTATGGGCAAGGGAATAGCTTTGGACTTGACGGGAGTTACCCACAAACCGAAAGTTATTAACGAGCCAGGGAAAGTATCAGTAGAGGTGGCAATGAAGATAAATGCCGCATTGATGGAGCTGTGTAAGAAGGTTATGGGCGCCGATGACGCAGCAACTAAGACAAAATTCTTCTTGTACGTGATGCAGATTGCTTGCACGTTCTTTACATCGTCTTCGACGGAGTTCAAAGAGTTTGACTACATAGAAACCGATGATGGAAAGAAGATATATGCGGTGTGGGTATATGATTGCATTAAACAAGCTGCTGCTTCGACGGGTTATGAAAACCCGGTAAGGCAGTATCTAGCGTACTTCACACCAACCTTCATCACGGCGACCCTGAATGGTAAACTAGTGATGAACGAGAAGGTTATGGCACAGCATGGAGTACCACCGAAATTCTTTCCGTACACGATAGACTGCGTTCGTCCGACGTACGATCTGTTCAACAACGACGCAATACTAGCATGGAATTTAGCTAGACAGCAGGCGTTTAGAAACAAGACGGTAACGGCCGATAACACCTTACACAACGTCTTCCAACTATTGCAAAAGAAGTAG

>GLRaV-1 1Z_BA

GAAATCTTTACCAACCCCGAGATGAATATCATCTTCGAACCACCAAAGGACATGGAGGTTTCGGTGGTAGTACCAACCGGACCCGGCTTGGTCACGCCGGCGGTGGCTACTGCAATTTCCACAGAATTAAAAAATTTATGTGCTGAAGTGATGGGTAATACAGATCAGAAAAGTCTTACAGACTTCTTCTTGGCGATGTTGCAATTGATGTTAACGTTTAGCACGCCACCAGA

>GLRaV-1 16Z_BA

GAAATCTTTACCAACCCCGAGATGAACATCATCTTTGAACCACCAAAAGAAATGGAGGTTTCGGTGGTAGTACCAACCGGGCCCGGCTTGGTTACACCAGCGGTGGCAACAGCTATATCAACAGAATTAAAAAATTTATGTGCCGAAGTTATGGGTAATACGGATCAGAAAAGTCTTACAGACTTCTTCTTGGCAATGTTACAATTGATGCTGACGTTTAGCACGTCACCAGA

>GLRaV-1 52B_BA

GAAATCTTTACCAAACCCGAGATGAATATTATCTTTGAACCACCAAAGGACATGGAAGTTTCGGTTGTGGTGCCGACCGGACCCGGCTTGGTGACTCCGGCAGTGGCAACAGCTATATCTACGGAATTAAAAAATTTATGTGCTGAAGTGATGGGTAACACTGATCAGAAAAGTCTCACAGATTTTTTCTTGGCAATGTTGCAATTAATGTTGACGTTTAGCACGTCACCAGA

>GLRaV-1 77B_BA

GAAATCTTTACCAAACCCGAGATGAATATTATCTTTGAACCACCAAAGGACATGGAAGTTTCGGTTGTAGTACCAACCAACACCGGCTTGGGTACACCAGCAAAGGCAACAGCAGTTTCAACAGAATTAAAAAATTTATGTGCCGAAGTTATGGGTAATACGGATCAGAAAAGTCTTACAGACTTCTTCTTGGCAATGTTACAATTGATGCTGACGTTTAGCACGTCACCAGA

>GFLV 5Z_BA

TTTTAAGATTGTTGTTAGGCTGCCCGCAAATGCCTTTACTGGGCTGACATGGGTCATGAGCTTTGATGCATACAATCGGATAACTAGTAGGATTACTACTAGTGCTGATCCTGTGTATACTTTGTCAGTTCCTCATTGGCTTATCCACCATAAGTCGGGCACATTTTCTTGTGAGATAGACTATGGAGAATTGTGTGGACATGCAATGTGGTTCAAAACCACAACATTTGAATCTCCAAGGTTACATTTTACATGCCTAACTGGTAATAATAAAGAGTTAGCAGCAGACTGGCAAGCTGTCGTTGAGTTGTATGCTGAATTGGAAGAGGCCACCTCTTTTCTTGGGAAACCTACTTTGGTTTTTGACCCGGGTGTTTTTAATGGCAAATTCCAATTCTTGACTTGCCCTCCCATATTCTTTGATCTAACAGCCGTCACGGCTCTCAGGAGTGCTGGGCTGACGCTGGGCCAAGTCCCAATGGTCGGCACTACCAAGGTTTATAACCTAAATAGCACCCTTGTGAGTTGTGTTCTAGGAATGGGAGGTACTATTAAAGGAAAGGTTCACATTTGTGCGCCAATCTTTTATAGTATTGTTTTATGGGTTGTTAGTGAGTGGAACGGGACCACTATGGATTGGAATGAGCTTTTTAAGTATCCCGGGGTGTATGTGGAAGAAGACGGAAGCTTTGAAGTTAAAATTCGTTCTCCATATCACCGAACGCCTGCTAGATTGCTTGCTGGTCAAAGTCAGAGGGATATGAGCTCTTTGAATTTTTATGCAATAGCAGGACCTATTGCTCCAACGGGTGAAACTGCACGACTTCCTGTTGTCGTGCAGATTGATGAAATCGAGCGCCCAGACCTCTCTTTGCCAAGTTTCGAAGATGATTATTTTGTGTGGGTGGATTTTTCTGAGTTCACTCTCGACAGAGAAGAAATTGAGATTGGTTCTCGTTTCTTTGATTTCACTTCAAGTACTTGCAAAGTGTCTATGGGAGAGAATCCGTTTGCTGCAATGATTGCTTGTCACGGGTTGCATAGTGGTGTATTAGACCTCAAATTACAATGGAGTCTGAACACCGAATTTGGCAAGAGCAGCGGGAGCATCACCGTTACGAAGCTGGTTGGTGATAAAGCCTTGGGCCTGGATGGACCTTCTCAAGTCTTTGCTATTCAAAAACTAGAGGGAACTACAGATTTG

>ArMV ArZ_BA

AGGGTCGCTTCTAGTACAGCTCCCGGTAGGGCTGCATGGATTTCAGAGCGTCGTAGTGCTCTGAGAAGGAGAGAGCAGGCTAACAGCCTGCAAGGCCTTGCTGCTCAAACAGACATGACATTTGAGCAGGCCAGAAACGCATATCTTGGTGCTGCTGATATGATCGAGCAAGGCTTGCCACTACTACCTCCTCTGAGAAATGCTTATGCACCTCGAGGTCTATGGAGGGGACCCTCTACCAGAGCCAACTTCACTTTGGATTTTAGGCTCAATGGCATCCCTACTGGGCAAAATACTCTCGAAATTTTGTATAACCCAGTAGCGGACGAGGAAATGGATGACTACCGTGACAGGGGTATGTCAGCGGTCGTGATTGATGCACTTGAAATTGCTATTAATCCTTTTGGAATGCCAGGCAATCCGACCGATCTTACTGTCGTGGCTACATATGGGCATGAGCGTAATATGGAACGTGCCTTTATTGGTTCCTCCTCAACTTTTCTCGGGAATGGGTTAGCGAGGGCCATTTTCTTTCCTGGTTTGCAATATAGTCAGGAAGAACCTAGGCGCGAATCTCTTATTCGCCTGTATGTAGCTTCCACTAATGCCACTGTTGATGCTGATTCCATTTTGGCGGCTATTAGTGTTGGTACTTTACGCCAGCACATTGGTTCGTTGCATAATAGGACGGTGGCCAGTTCTGTGCATGCTGCACAAGTGCAAGGCACTACCTTGAGGGCTACTATGATGGGTAACGCTGTCGTGGTATCTCCCGAGGGAAGTCTTGTTACTGGAACCCCTGAAGCTAACGTCCAAATGGGAGGTGGTTCAAGCATGCGAATGGTGGGTCCCTTGGCTTGGGAAAATGTTGAAGAACCTGGTCAAACTTTTACCATAAGAAACCGCTCTAGGTCCATGCGAGTGGATCGAAATGCTGATGTTGGAGTTGCTCTTCCTCGGAT
